# Granulopoiesis Requires Increased C/EBPα Compared to Monopoiesis, Correlated with Elevated *Cebpa* in Immature G-CSF Receptor versus M-CSF Receptor Expressing Cells

**DOI:** 10.1371/journal.pone.0095784

**Published:** 2014-04-21

**Authors:** Ou Ma, SunHwa Hong, Hong Guo, Gabriel Ghiaur, Alan D. Friedman

**Affiliations:** 1 Division of Pediatric Oncology, Johns Hopkins University, Baltimore, Maryland, United States of America; 2 Division of Hematologic Malignancies, Johns Hopkins University, Baltimore, Maryland, United States of America; Albert Einstein College of Medicine, United States of America

## Abstract

C/EBPα is required for the formation of granulocyte-monocyte progenitors; however, its role in subsequent myeloid lineage specification remains uncertain. Transduction of murine marrow with either of two *Cebpa* shRNAs markedly increases monocyte and reduces granulocyte colonies in methylcellulose or the monocyte to neutrophil ratio in liquid culture. Similar findings were found after marrow shRNA transduction and transplantation and with *CEBPA* knockdown in human marrow CD34^+^ cells. These results apparently reflect altered myeloid lineage specification, as similar knockdown allowed nearly complete 32Dcl3 granulocytic maturation. *Cebpa* knockdown also generated lineage-negative blasts with increased colony replating capacity but unchanged cell cycle parameters, likely reflecting complete differentiation block. The shRNA having the greatest effect on lineage skewing reduced *Cebpa* 3-fold in differentiating cells but 6-fold in accumulating blasts. Indicating that *Cebpa* is the relevant shRNA target, shRNA-resistant C/EBPα-ER rescued marrow myelopoiesis. *Cebpa* knockdown in murine marrow cells also increased *in vitro* erythropoiesis, perhaps reflecting 1.6-fold reduction in *PU.1* leading to GATA-1 derepression. Global gene expression analysis of lineage-negative blasts that accumulate after *Cebpa* knockdown demonstrated reduction in *Cebpe* and *Gfi1*, known transcriptional regulators of granulopoiesis, and also reduced *Ets1* and *Klf5*. Populations enriched for immature granulocyte or monocyte progenitor/precursors were isolated by sorting Lin^−^Sca-1^−^c-Kit^+^ cells into GCSFR^+^MCSFR^−^ or GCSFR^−^MCSFR^+^ subsets. *Cebpa*, *Cebpe*, *Gfi1*, *Ets1*, and *Klf5* RNAs were increased in the c-Kit^+^GCSFR^+^ and *Klf4* and *Irf8* in the c-Kit^+^MCSFR^+^ populations, with *PU.1* levels similar in both. In summary, higher levels of C/EBPα are required for granulocyte and lower levels for monocyte lineage specification, and this myeloid bifurcation may be facilitated by increased *Cebpa* gene expression in granulocyte compared with monocyte progenitors.

## Introduction

CCAAT/enhancer binding protein α (C/EBPα) is a basic region-leucine zipper transcription factor expressed within granulocytic and monocytic myeloid cells during hematopoiesis; C/EBPα is the predominant C/EBP protein in immature myeloid cells [1], [2]. Newborn C/EBPα (−/−) mice lack granulocytes but retain monocytes; however, marrow from adult C/EBPα (flox/flox);Mx1-Cre mice exposed to pIpC to induce *Cebpa* gene deletion have markedly reduced numbers of granulocyte-monocyte progenitors (GMP), leading to impairment of both granulopoiesis and monopoiesis, with increased numbers of preceding common myeloid progenitors (CMP) [3], [4]. In addition, exogenous C/EBPα directs granulocytic maturation of the U937, HL-60, or 32Dcl3 myeloid cell lines but induces monocytic maturation of murine marrow myeloid progenitors or lymphoid cells [2], [5]–[8]. The role of C/EBPα beyond the GMP in directing myeloid lineage specification thus remains uncertain.

To gain further insight into the regulation of myelopoiesis by C/EBPα, we have investigated the consequences of reducing but not eliminating C/EBPα expression. We find that two different *Cebpa* shRNAs impair murine marrow granulopoiesis and enable increased monopoiesis. *Cebpa* knockdown also led to accumulation of an immature population apparently unable to commit to either lineage, with increased *in vitro* growth and replating capacity, a preleukemic phenotype. *Cebpa* RNA was reduced 3-fold in the bulk population that retains myeloid differentiation capacity but 6-fold in lineage-negative cells unable to mature along either lineage. In addition to use of two independent shRNAs, we further support the conclusion that *Cebpa* is the relevant shRNA target by demonstrating that shRNA-resistant C/EBPα-ER overcomes the block to marrow cell myeloid development. Supporting the idea that *Cebpa* knockdown blocks granulocyte lineage commitment and not simply granulocytic maturation, we show that 5-fold *Cebpa* reduction in the 32Dcl3 cells line allows maturation to the metamyelocyte/band stage in response to G-CSF. *Cebpa* knockdown also unexpectedly increased marrow cell erythropoiesis, potentially reflecting reduced *Sfpi1/PU.1* expression and thereby GATA-1 derepression [9], [10].

To gain insight into the mechanism underlying their impeded myelopoiesis, we conducted global RNA expression analysis of control versus *Cebpa* knockdown lineage-negative cells, revealing multiple changes including reduction in *Cebpe* and *Gfi1*, known C/EBPα targets and regulators of granulopoiesis, and also reduced *Ets1* and *Klf5*. Having observed reduced granulopoiesis but preserved monopoiesis upon partial *Cebpa* knockdown, we also sought to determine whether *Cebpa* RNA levels are actually elevated in marrow granulocyte compared with monocyte progenitors/precursors. Immature Lin^−^Sca-1^−^c-Kit^+^ cells were sorted into populations exclusively expressing the G-CSF receptor (GCSFR) or the M-CSF receptor (MCSFR). CFU-G were mainly found in the former and CFU-M in the latter subset, and there was significantly higher *Cebpa* mRNA levels in the c-Kit^+^GCSFR^+^ population. Notably, *Cebpe*, *Gfi1*, *Ets1*, and *Klf5* RNAs were also increased in this population and *Klf4* and *Irf8* in the c-Kit^+^MCSFR^+^ subset, with *PU.1* similar in both. The relevance of our findings to normal and malignant myelopoiesis will be further discussed.

## Methods

### Ethics Statement

This study was carried out in strict accordance with the recommendations in the Guide for the Care and Use of Laboratory Animals of the National Institutes of Health. The protocol (M013M116) was approved by the Johns Hopkins University Animal Care and Use Committee. All efforts were made to minimize suffering. Use of human marrow samples from donors who gave written informed consent was approved by the Johns Hopkins University Institutional Review Board.

### shRNAs and Plasmids

To target murine *Cebpa*, we utilized shRNAs TRCN9502–9504 (B9–B11) in the pLKO.1-Puro lentiviral vector (Open Biosystems). Human *CEBPA* was targeted with TRCN7305–6 (G5, G6). Empty vector and mammalian shRNA SHC002 non-targeting vector (NTV, Sigma Aldrich) were employed as controls. To rescue C/EBPα expression we modified the C/EBPα-ER cDNA to be resistant to B9 shRNA by changing the targeted site AGC-CGA-GAT-AAA-GCC-AAA-CAG to *TCT*-*A*G*G*-GA*C*-AA*G*-GC*T*-AA*G*-CAG, with synonymous changes italicized.

### Murine Marrow Culture, Transduction, FACS Analysis, and Transplantation

Marrow isolated from 8–16 week old C57BL/6 female mice was subjected to red cell lysis with NH_4_Cl, and the cells were lineage-depleted using biotin-conjugated B220, Gr-1, Mac-1, Ter119, and CD3 mouse Lineage Cocktail (BD Pharmingen), anti-biotin microbeads, and MACS columns (Miltenyi Biotec). The cells were then cultured in Iscove’s modified Dulbecco medium (IMDM) with 10% heat-inactivated fetal bovine serum (HI-FBS) containing 50 ng/mL murine stem cell factor (SCF), 100 ng/mL murine FLT3 ligand (FL), and 10 ng/mL murine thrombopoietin (TPO, Peprotech) for 24 hrs. Lentiviral particles were generated by transient transfection of 293T cells with plasmids at a ratio of 3.75 µg pLKO.1 vector: 5 µg pCMV-ΔR8.91: 1.25 µg pMD.G(VSV.G) per 100 mm dish using 20 µL Lipofectamine 2000 (Invitrogen). Lentiviral supernatants collected at 48 hr were concentrated with AmiconUltra filters (Millipore). Retroviral particles were packaged similarly at a ratio of 8 µg MIG or pBabe vector: 2 µg pkat2ecopac. The cells were transduced by spinoculation at 1500 g for 2 hours after addition of lentiviral supernatant alone or with retroviral supernatant and 1 µg/mL Polybrene. Co-culture with viral supernatant and 0.5 µg/mL Polybrene was then continued for 2 days, followed by addition of 2.5 µg/mL puromycin for 2 additional days. Viable cells were collected using Lympholyte-M polysucrose (Cedarlane Labs) and transferred to IMDM with 10% HI-FBS with 10 ng/mL murine IL-3, 10 ng/mL murine IL-6, and 50 ng/mL murine SCF in liquid culture or to Methocult M3231 (StemCell Technologies) containing methylcellulose with IMDM, HI-FBS, and these same cytokines at 1E4 cells/mL for myeloid colony analysis. Erythroid colonies were obtained by plating 1.6E5 cells/mL in Methocult 3120 (1% final concentration) with IMDM, 2 mM glutamine, 55 nM β-mercaptoethanol, 10% plasma-derived serum (Animal Technologies), 20% BIT (Stem Cell Technologies), 5% PFHM-II (Invitrogen), and 2 U/mL (20 ng/mL) murine erythropoietin (EPO). Colony forming units (CFUs) were enumerated 7 days later. Colony replating was done weekly also at 1E4 cells/mL.

Cells were subjected to morphologic analysis via cytospin and Wright-Giemsa staining, to cell cycle analysis via propidium iodide (PI) staining after fixation in 70% methanol and treatment with DNase-free RNase, and to surface marker analysis via fluorescent activated cell sorting (FACS) using PE-anti-Mac-1, FITC-anti-Gr-1, PerCP-Cy5.5-anti-Sca-1, APC-anti-c-Kit, and PE-anti-Ter119. For CMP/GMP/MEP analysis, cells were stained with biotin-anti-lineage markers, PerCP-Cy5.5-streptavidin, PE-Cy7-anti-Sca-1, APC-anti-c-Kit, FITC-anti-CD34, and PE-anti-FcγR. Photomicrographs were taken using a Zeiss Axiophot microscope (Carl Zeiss), a Kontron Electronik Progress 3012 camera (Kontron), and a 63X/1.40 NA oil objective. Viable cell counts were enumerated using Trypan blue dye and a hemocytometer.

For *in vivo* analysis, 8E5 puromycin-resistant CD45.2^+^ cells combined with 2E5 CD45.1^+^ nucleated marrow cells were transplanted via tail vein injection into syngeneic CD45.1 recipients irradiated to 950 cGy. At day 28 marrow was analyzed by FACS and sorted for CD45.2^+^CD45.1^−^ cells for CFU assay via use of a FACSAria II cell sorter (BD Biosciences).

### Human Marrow Culture, Transduction, and FACS Analysis

Cryopreserved human bone marrow CD34^+^ cells were obtained from the Johns Hopkins cell bank. Cells were placed directly in serum-free Stemline II Hematopoietic Stem Cell Expansion Medium (Sigma-Aldrich) with 100 ng/mL FLT3 ligand, 100 ng/mL human SCF, and 20 ng/mL human thrombopoietin (TPO) and recovered for 2 days, followed by transduction with lentiviral vectors in the presence of 2 µg/ml Polybrene for 2 consecutive days. On the third day, transduced cells were subject to 2 µg/ml puromycin selection for 2 days, and viable cells were separated by density gradient centrifugation (Ficoll-Paque Plus; GE Healthcare Life Sciences) at 1,500 rpm for 30 minutes, and cultured either in methylcellose (Methocult H4230, StemCell Technologies) with IMDM at 2E4 cells/mL or in liquid culture with RPMI and 10% HI-FBS at 2E5 cells/ml, in either case with 50 ng/ml human GM-CSF. Myeloid colonies were enumerated on day 14. For FACS, cells were stained with hIgG Fc Receptor Block (Biolegend), FITC-anti-CD14, and PE-anti-CD15.

### 32Dcl3 Cell Culture and Transduction

32Dcl3 cells [11] were maintained in IMDM with 10% HI-FBS with 1 ng/mL murine IL-3. To induce differentiation, cells were washed twice with PBS and transferred to IMDM with 10% HI-FBS and 20 ng/mL human G-CSF (Amgen). 32Dcl3 cells were transduced for 2 days by addition of lentiviral or retroviral supernatant in the presence of 4 µg/mL Polybrene, followed by selection with 2 µg/mL puromycin or 1.2 mg/mL G418 (total). Subclones were obtained by limiting dilution. G-CSF receptor (GCSFR) surface expression was evaluated using biotin-G-CSF and streptavidin-APC +/−100-fold excess G-CSF. Biotin G-CSF was generated using human G-CSF (Amgen) and a biotinylation kit (Pierce).

### Isolation of Populations Enriched for CFU-G Or CFU-M

Marrow isolated from C57BL/6 mice was subjected to red cell lysis with NH_4_Cl and then stained with FITC-anti-Lineage (Lin) Markers (CD3, B220, Ter119, Gr-1, Mac-1; BioLegend). PerCP-Cy5.5-anti-c-Kit (2B8; BioLegend), PE-Cy7-anti-Sca-1 (D7; eBioscience), PE-anti-MCSFR (AFS98; BioLegend), biotin-G-CSF, and streptavidin-APC. The Lin^−^c-Kit^+^Sca-1^−^GCSFR^+^MCSFR^−^ and Lin^−^c-Kit^+^Sca-1^−^GCSFR^−^MCSFR^+^ populations were then isolated by flow cytometry. GMPs were isolated by flow cytometry as described [12].

### Western Blot and RNA Analysis

For Western blotting, samples in Laemmli sample buffer (LSB) corresponding to 5E5 cells were subjected to PAGE using 10% gels and transferred to Hybond-P membrane (Amersham). Primary antibodies used were C/EBPα (14AA, Santa Cruz Biotechnology) and β-actin (AC-15; Sigma-Aldrich). After addition of HRP-conjugated secondary antibody and subsequent washes, a signal was generated using HyGlo chemiluminescence reagents (Denville Scientific) and detected by autoradiography.

RNA was prepared from 1E6–1E7 cells using the NucleoSpin RNA II kit, with use of RNase-free DNase (Machery-Nagel). First strand cDNA was prepared using AMV reverse transcriptase (Promega) and oligo(dT) primer. Quantitative PCR was carried out using 50 ng of each cDNA using iQ SYBR Green supermix (Bio-Rad). Oligonucleotides used are listed in Table S1.

### Global Gene Expression Analysis

Mouse WG-6 v2.0 Expression BeadChips (Illumina) were used for microarray hybridizations. Total RNA was prepared using the NucleoSpin RNA II kit. RNA quality was confirmed via Nanodrop-1000 spectrometric analysis and via a Bioanalyzer (Agilent Technologies). 500 ng total RNA from each sample was amplified and labeled using the Illumina TotalPrep RNA Amplification Kit with oligo(dT) priming (Ambion). 750 ng biotin-labeled cRNA was combined with hybridization buffer and hybridized to the array at 58°C for 16–20 hours. After hybridization, the array was washed with buffer at 55°C and blocked at room temperature. Bound, biotinylated cRNA was stained with streptavidin-Cy3 and then washed. Dried arrays were scanned with the iScan System, and data with quantile normalization was exported from GenomeStudio v2011.1 Gene Expression Module 1.9.0 and imported to GeneSpring. Differentially expressed genes with >1.5-fold change were subjected to pathway analysis using Ingenuity Systems software. Analysis of fold-change between paired samples was conducted without subtraction of background signal. Primary microarray data is available from GEO (accession number GSE55760).

### Statistical Analysis

Means and standard deviations are shown. The Student *t* test was used for statistical comparisons. Band intensities were quantified using National Institutes of Health ImageJ 1.38 software.

## Results

### *Cebpa* Knockdown Reduces CFU-G While Increasing CFU-M and a Population of Immature Blasts

**Figure 1:**
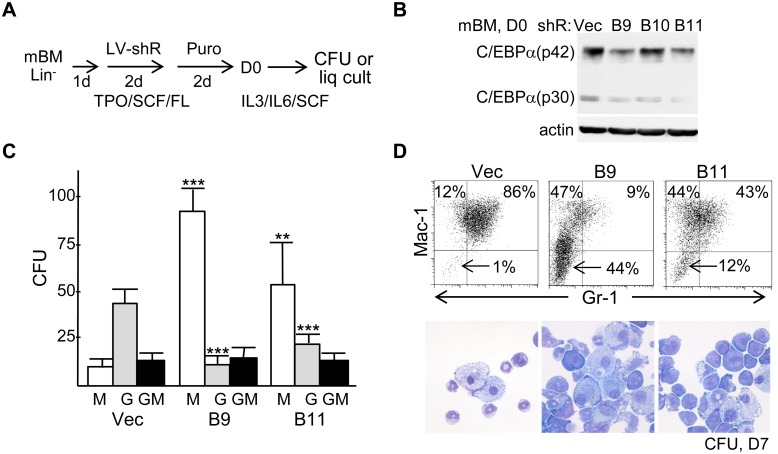
*Cebpa* knockdown increases murine marrow cell monopoiesis relative to granulopoiesis and generates a blast population. **A**) Diagram of marrow transduction protocol. **B**) Western blot for C/EBPα and β-actin after transduction and puromycin selection with the pLKO.1 vector (Vec) or with *Cebpa* shRNAs B9 or B11. **C**) Number of CFU-M, CFU-G, or CFU-GM per 1E4 cells plated in methylcellulose culture with IL-3, IL-6, and SCF (n = 6–9). **D**) FACS analysis for Mac-1 and Gr-1 on pooled CFUs. Arrows indicate immature, lineage-negative cells (top). Morphology of pooled CFU cells (bottom). *p<0.05, **p<0.01, ***p<0.001.

Murine lineage-negative marrow cells were transduced with pLKO.1 lentiviral vectors expressing one of several *Cebpa* shRNAs (B9, B10, or B11) or the empty pLKO.1 vector for 2 days followed by puromycin selection for 2 additional days. This was done in TPO, SCF, and FL, cytokines that maintain the majority of cells undifferentiated [13]. Viable cells were then transferred to IL-3, IL-6, and SCF to allow myeloid maturation (Figure 1A). The B9 or B11 shRNAs reduced C/EBPα protein 2-fold relative to the pLKO.1 vector, based on image scanning, in cells collected on D0 directly after drug selection (Figure 1B). B9 or B11 reduced CFU-G while increasing CFU-M relative to empty vector (Figure 1C). Typical murine CFU-G, CFU-M, and CFU-GM colony morphologies are shown (Figure S1A). FACS analysis of pooled CFUs to enumerate Mac-1^+^Gr-1^+^ granulocytes and Mac-1^+^Gr-1^−^ monocytes confirmed reduced granulopoiesis relative to monopoiesis (Figure 1D, top). We previously demonstrated that Mac-1^+^Gr-1^−^ cell in similar analyses express both CD115/MCSFR and F4/80, confirming their identity as monocytes [12]. FACS analyses herein also revealed accumulation of an immature Mac-1^−^Gr-1^−^ population, evident in this representative analysis as 1% of vector-transduced but 44% or 12% of B9 or B11-transduced CFU cells, respectively (arrows). Morphologic analysis revealed both macrophages and neutrophils after vector-transduction, but predominantly macrophages and cells with blast morphology in CFUs derived from B9- or B11-transduced marrow (Figure 1D, bottom). Together, these data indicate that graded C/EBPα levels guide myeloid lineage specification and that sufficient reduction of C/EBPα prevents progression along either lineage.

**Figure 2:**
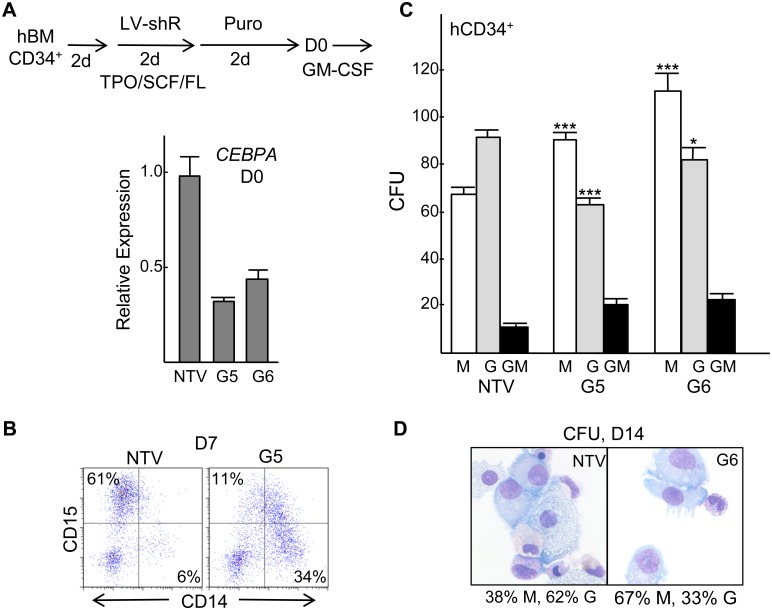
*CEBPA* knockdown increases human marrow cell monopoiesis relative to granulopoiesis. **A**) Diagram of marrow transduction protocol (top) and *CEBPA* mRNA expression normalized to β-Actin on D0 after transduction with non-targeting vector (NTV) or *CEBPA* shRNAs G5 or G6. **B**) FACS analysis for the CD14 (monocytic) and CD15 (granulocytic) markers at D7 for liquid cultured cells. **C**) Number of CFU-M, CFU-G, and CFU-GM per 5E4 cells plated (n = 3). **D**) Representative morphology of pooled CFU cells. Note large monocytes with abundant basophilic cytoplasm and smaller granulocytic cells with eosinophilic cytoplasm and granules. The proportion of monocytes (M) and granulocytes (G) amongst 100 cell counted are shown.

Human bone marrow CD34^+^ cells were transduced in TPO/FL/SCF with non-targeting lentiviral vector (NTV) or with vectors expressing human *CEBPA* shRNAs G5 or G6, followed by puromycin selection in these same cytokines and then transfer on D0 to GM-CSF to induce myeloid differentiation, as diagrammed (Figure 2A, top). Quantitative RT-PCR analysis demonstrated approximately 3-fold *CEBPA* RNA knockdown by G5 and 2.5-fold knockdown by G6 on D0 (Figure 2A, bottom). After 7 days in liquid culture, cells transduced with NTV or G5 were subjected to CD14/CD15 FACS, with G5 transduction reducing CD15^+^ granulocytic cells and increasing CD14^+^ monocytic cells (Figure 2B). *CEBPA* knockdown by either of two shRNAs also increased CFU-M and reduced CFU-G relative to NTV, without diminishing total CFU numbers (Figure 2C). Morphologies of typical human CFUs obtained are shown (Figure S1B). Pooled CFUs transduced with NTV or G6 were cytospun, stained with Wright-Giemsa stain, and myeloid cells were enumerated, revealing a reduced proportion of granulocytes and a corresponding increased proportion of monocytes (Figure 2D).

### *Cebpa* Knockdown Increases Myeloid Colony Replating

**Figure 3:**
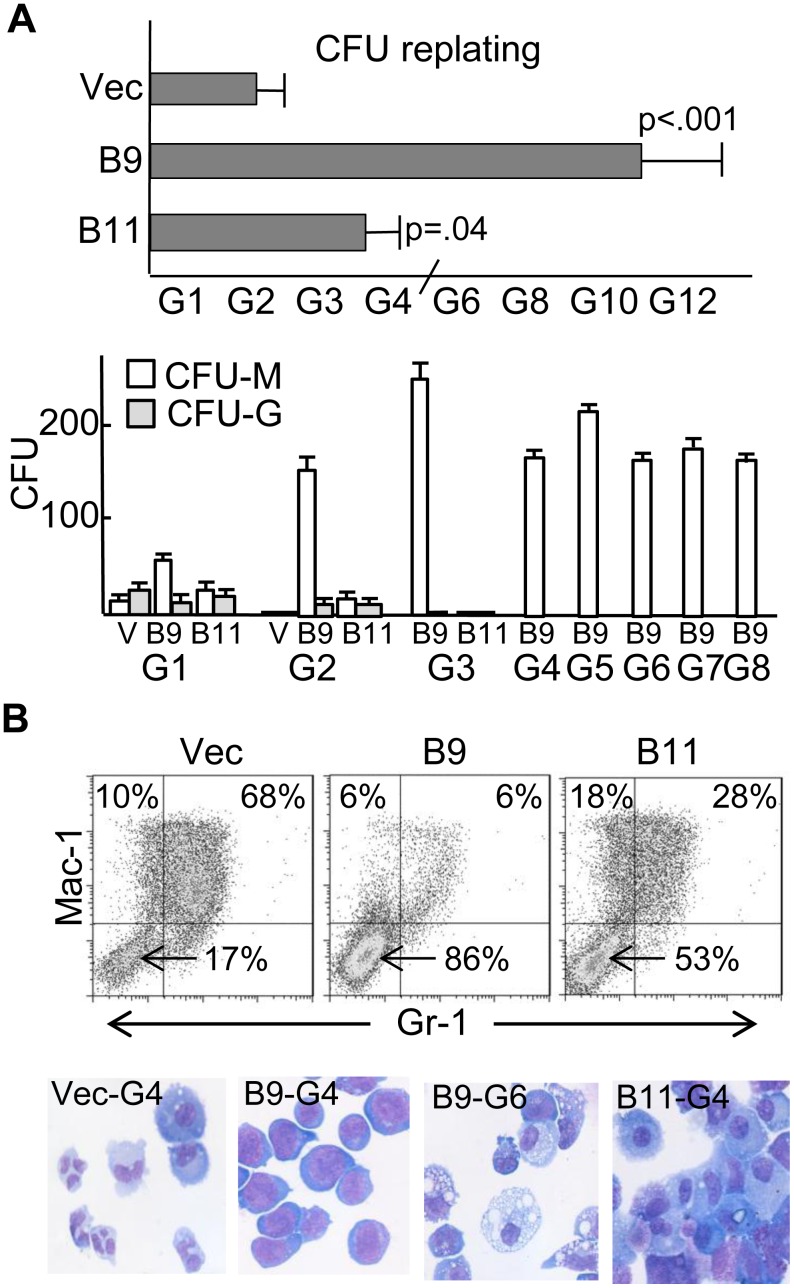
*Cebpa* knockdown increases myeloid colony replating. **A**) Myeloid CFUs obtained after transduction with the pLKO.1 vector or with *Cebpa* shRNAs B9 or B11 were replated each 7 days. The average number of generations for which CFUs were obtained is plotted (top, n = 4–5). G4 indicates that initial CFUs could be replated 3 times to yield CFUs. CFU-G and CFU-M obtained at each generation from a typical experiment done in triplicate, 1E4 cell/plate (bottom). **B**) FACS analysis for Mac-1 and Gr-1 from G3 CFUs. Arrows indicate immature, lineage-negative cells (top). Morphology of G4 and G6 CFUs from the same experiment (bottom).

To determine whether the blastic, lineage-negative cells that accumulate after transduction of murine marrow with *Cebpa* shRNAs have increased proliferative capacity, CFUs were replated weekly. CFUs derived from pLKO.1-transduced marrow cells lost replating capacity after an average of 2.2 generations, and CFUs expressing a non-targeting shRNA stopped replating after 2.0 generations (not shown). In contrast, CFUs obtained after B11-transduction replated an average of 3.75 generations (p = 0.04), and those obtained from B9-transduced cells replated for at least 8 generations (p<0.001, Figure 3A, top), with CFU-M predominating (Figure 3A, bottom). Although neither B9- nor B11-transduced cells gained cytokine independence, in one experiment we continued replating B9-transduced cells for 16 generations before stopping. FACS analysis of third generation CFUs demonstrated strong accumulation of Mac-1^−^Gr-1^−^ cells in both the B9 and B11 cultures, with shift of the remaining cells towards monopoiesis (Figure 3B, top). Morphologic analysis demonstrated macrophages and blasts but no granulocytes in B9 or B11 CFUs (Figure 3B, bottom).

### *Cebpa* Knockdown Impairs Granulopoiesis and Increases Monopoiesis in Liquid Culture without Altering the Cell Cycle

**Figure 4:**
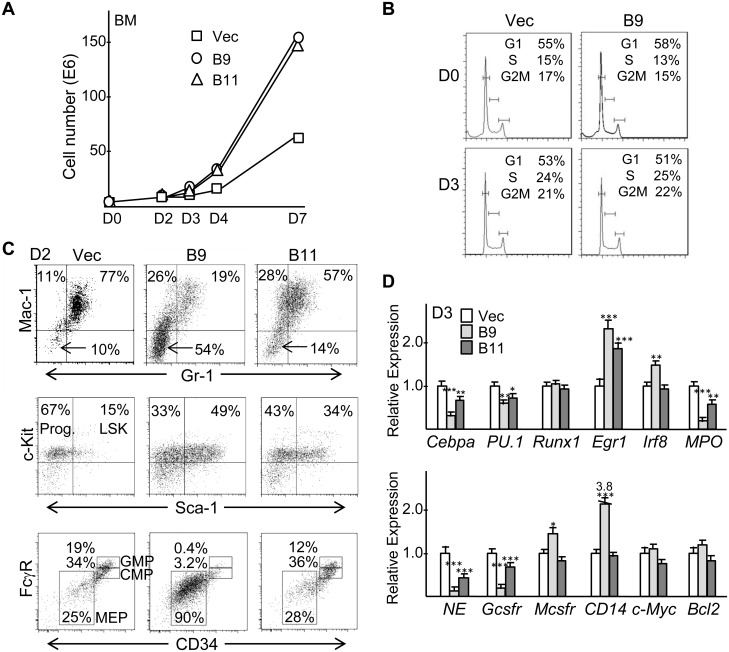
*Cebpa* knockdown reduces murine granulopoiesis relative to monopoiesis and increases immature blasts in liquid culture. **A**) Transduced, puromycin selected marrow cells were placed in liquid culture with IL-3, IL-6, and SCF on D0 and viable cell counts were enumerated on days 0, 2, 3, 4, and 7. Representative data is shown. **B**) Cell cycle analysis was conducted using PI staining on D0 or D3. **C**) FACS analysis for Mac-1 and Gr-1 on D2. Arrows indicate immature, Mac-1^−^Gr-1^−^ cells (top). FACS analysis of Mac-1^−^Gr-1^−^ cells stained for Sca-1 and c-Kit (middle). FACS analysis of D2 cells for FcγR and CD34, gating on Lin^−^Sca-1^−^c-Kit^+^ progenitors (bottom). Representative data are shown. **D**) Total cellular RNAs collected on day 3 (D3) after lentiviral transduction with the pLKO.1 vector or with *Cebpa* shRNAs B9 or B11, puromycin selection, and transfer to IL-3, IL-6, and SCF were subjected to quantitative RT-PCR for indicated mRNAs (n = 3).

To investigate the early effects of *Cebpa* knockdown, transduced marrow cells were placed in liquid culture with IL-3, IL-6, and SCF. B9- or B11-transduced cells expanded more rapidly, as shown for a representative experiment (Figure 4A). Although exogenous C/EBPα inhibits G1 to S cell cycle progression in myeloid cells [5], cell cycle analysis did not uncover a significant difference in the proportion of cells in the G1, S, or G2/M cell cycle phases due to *Cebpa* knockdown, either on D0 or D3 after completion of puromycin selection, and there was no difference in the small number of sub-G1 apoptotic cells (Figure 4B).

FACS analysis revealed that as early as D2 there was reduction in the proportion of granulocytes, increased monocytes, and accumulation of immature Mac-1^−^Gr-1^−^ cells; these changes were most evident with B9 but also occurred with B11 *Cebpa* shRNA transduction (Figure 4C, top). As a control, we transduced marrow cells with non-targeting shRNA in comparison to empty pLKO.1 or the B9 shRNA. After 2 days in liquid culture FACS analysis demonstrated near equivalence of the two control cultures and again showed reduced granulocytes, increased monocytes, and striking accumulation of Mac-1^−^Gr-1^−^ cells with B9 but not the two control vectors (Figure S2).

FACS analysis also revealed that the majority of Mac-1^−^Gr-1^−^ (Lin^−^) cells are Lin^−^Sca-1^−^c-Kit^+^ progenitors or Lin^−^Sca-1^+^c-Kit^+^ (LSK) stem cells, with the stem cell subset increased by *Cebpa* knockdown (Figure 4C, middle). When the Lin^−^Sca-1^−^c-Kit^+^ progenitor cells were further analyzed using FcγR and CD34 antibodies, the B9 and B11 cultures had diminished GMP relative to CMP in comparison to the control culture, consistent with results obtained upon deletion of the *Cebpa* genes in adult mice [4], and unexpectedly there was increased megakaryocyte/erythroid progenitors (MEP) with B9 (Figure 4C, bottom).

To further confirm that *Cebpa* knockdown reduces granulopoiesis while increasing monopoiesis, total cellular RNAs prepared from vector-, B9-, of B11-transduced marrow cells on D3 after transfer to IL-3, IL-6, and SCF were analyzed for several myeloid transcription factors and lineage markers (Figure 4D). *Cebpa* was reduced 3-fold by B9 and 2-fold by B11. Even though increased *PU.1* levels favor monopoiesis, *PU.1* mRNA was reduced 1.6-fold by B9 or B11. *Runx1* was unaffected. *Egr1* and *Irf8*, encoding proteins that direct monopoiesis, were increased. The granulocytic markers *Mpo*, *Ela2*/*NE*, and *Csf3r*/*Gcsfr* were reduced, more prominently by B9 compared with B11, whereas B9 but not B11 led to increased expression of the monocytic markers *Csf1r*/*Mcsfr* and *Cd14*. C/EBPα activates the gene encoding c-Myc via DNA-bound E2F1 and the gene encoding Bcl-2 via DNA-bound NF-κB p50 [14], [15]; however, B9 had no effect and B11 only minimal effect on expression of their corresponding mRNAs.

### *Cebpa* Knockdown Impairs Granulopoiesis and Increases Monopoiesis *In vivo*

**Figure 5:**
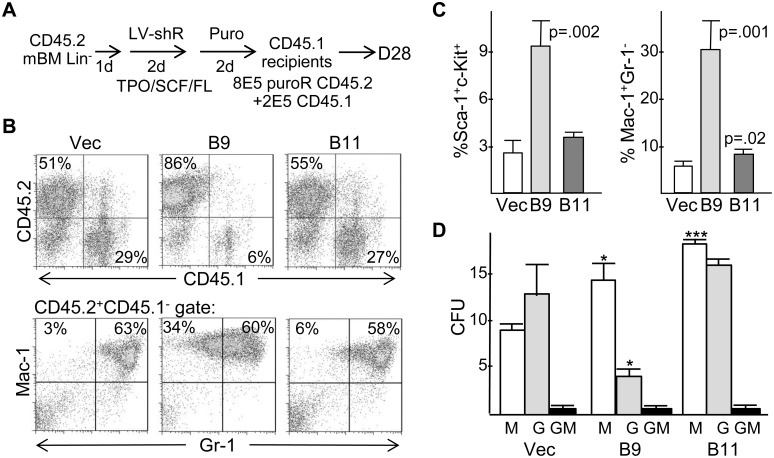
*Cebpa* knockdown reduces murine granulopoiesis and increases monopoiesis and immature cells *in vivo*. **A**) Diagram of transduction and transplantation protocol using the pLKO.1 vector or *Cebpa* shRNAs B9 or B11. **B**) Marrow cells isolated 28 days after transplantation (D28) were subjected to CD45.2/CD45.1 or Mac-1/Gr-1 FACS analysis. Representative data are shown. **C**) Summary of Sca-1/c-Kit and Mac-1/Gr-1 FACS analyses (vector and B9, n = 4; B11, n = 3). **D**) On day 28, CD45.2^+^CD45.1^−^ marrow cells isolated by flow cytometry were plated in methylcellulose with IL-3, IL-6, and SCF. The number of CFU-M, CFU-G, or CFU-GM obtained per 1E4 cells plated is shown (n = 3).

To evaluate the effect of *Cebpa* knockdown *in vivo*, 8E5 transduced CD45.2^+^ marrow cells were transplanted with 2E5 CD45.1^+^ carrier cells into lethally irradiated CD45.1^+^ recipient mice (Figure 5A). Compared with vector, marrow reconstituted with B9-transduced cells had a reproducibly higher proportion of CD45.2^+^ cells on day 28 (Figure 5B, top panels), 82+/−7% versus 41+/−11% (p = 0.002). In addition, marrow derived from B9-transduced cells had >3-fold increased Sca-1^+^c-Kit^+^ cells, averaging 9% (Figure 5C, left). These data indicate that *Cebpa* knockdown leads to a population of immature progenitors that expand over a 4 week period *in vivo*, paralleling the increased *in vitro* replating capacity of similarly transduced cells. This expanded, immature population was transient, as only 2 of 7 mice euthanized 20–24 weeks after transplant with B9-transduced marrow had >10% CD45.2^+^CD45.1^−^ marrow cells, and only 0.8% or 1.7% of these cells were Sca-1^+^c-Kit^+^ (not shown).

*Cebpa* knockdown also increased the proportion of marrow Mac-1^+^Gr-1^−^ monocytes relative to Mac-1^+^Gr-1^+^ granulocytes on day 28 after transplantation, with B9 having a greater effect than B11 (Figure 5B, bottom panels and 5C, right). This increase was no longer apparent in the two B9-transduced mice with >10% CD45.2^+^ cells at weeks 20–24 (not shown), further indicating that *Cebpa* knockdown affected the differentiation of a transient myeloid progenitor. Providing further support for an *in vivo* effect of *Cebpa* knockdown on myeloid specification, CD45.2^+^ marrow cells sorted from transplanted mice on day 28 were assessed for formation of myeloid CFUs in methylcellulose (Figure 5D). Both B9 and B11 increased CFU-M numbers and the CFU-M to CFU-G ratio, with B9 having a greater effect. Neither B9 nor B11 significantly altered the proportion of CD45.2^+^ cells expressing the erythroid marker Ter119, either on day 28 or at weeks 20–24 (not shown).

### Partial *Cebpa* Knockdown Allows Nearly Complete 32Dcl3 Granulocytic Maturation

**Figure 6:**
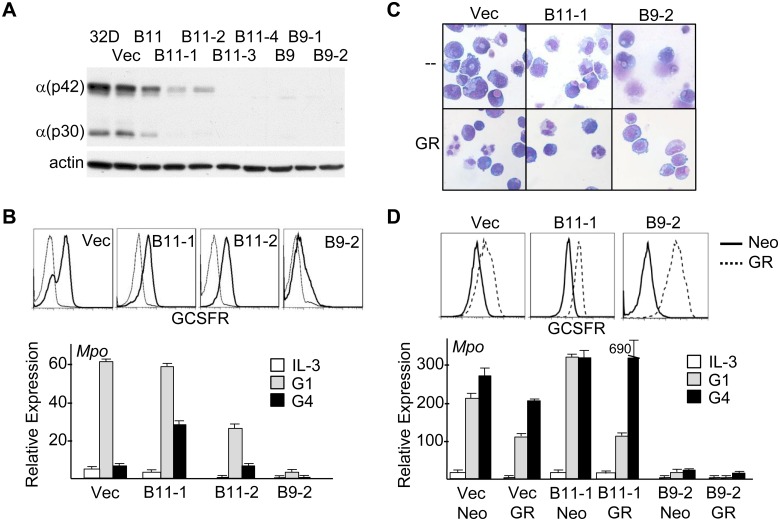
32Dcl3 cells with partial *Cebpa* knockdown retain capacity for nearly complete granulocytic maturation. **A**) Parental 32Dcl3 cells, pools of 32Dcl3 cells stably transduced with the pLKO.1 vector (Vec) or *Cebpa* B9 or B11 shRNAs, or subclones of the B9 or B11 pools were subjected to Western blotting for C/EBPα and β-actin. **B**) Indicated 32Dcl3 lines were subjected to FACS analysis for GCSFR using biotin-G-CSF in the absence (heavy line) or presence (light line) of excess unbiotinylated G-CSF; x-axis is log scale (top). RNAs prepared from indicated 32Dcl3 lines cultured in IL-3 or after one or four days in G-CSF (G1, G4) were subjected to quantitative RT-PCR for *Mpo* (bottom, n = 3). **C**) Vector, B11-1, or B9-2 cells cultured in GCSF were subjected to Wright-Giemsa staining on the days of maximal differentiation, G6, G8, and G4 respectively (top row). Vector/GR, B11-1/GR, or B9-2/GR, G14, G14, and G6, respectively, were analyzed similarly (bottom row). **D**) Pools of vector, B11-1, or B9-2 lines transduced with pBabeNeo or pBabeNeo-GCSFR were subjected to FACS analysis for GCSFR (top). RNAs prepared from these lines in IL-3 or G-CSF were analyzed for *Mpo* expression (bottom, n = 3).

Parental 32Dcl3 cells, pooled vector, B9 or B11 transductants, and several subclones were analyzed for C/EBPα expression by Western blotting (Figure 6A). Within the pools, B11 modestly and B9 almost completely reduced C/EBPα levels. Subclones B11-1 and B11-2 have 5-fold reduced C/EBPα protein, based on image scanning, while B11-3, B11-4, B9-1, and B9-2 have essentially absent C/EBPα. A similar pattern was seen on RNA analysis (not shown). The *GCSFR* promoter is activated by C/EBPα [16], and GCSFR surface expression is reduced modestly in B11-1 or B11-2 cells and more extensively in the B9-2 subclone (Figure 6B, top). These three subclones and vector-transduced cells were transferred to G-CSF and monitored for morphologic maturation and induction of the *Mpo* differentiation marker (Figure 6B, bottom and 6C, upper row). *Mpo* was induced fully in B11-1 cells and at mildly reduced levels in B11-2 cells, but only minimally in the B9-2 subclone, which did not survive past day 4 in G-CSF. Vector cells produced mature neutrophils; B11-1 cells, and B11-2 cells (not shown), matured to the metamyelocyte/band stage; B9-2 cells did not show any granulocytic maturation. B11-1 or B9-2 32Dcl3 cells transduced with pBabeNeo-GCSFR demonstrated rescue of GCSFR expression (Figure 6D, top). B11-1/GR cells now demonstrated full neutrophil maturation in G-CSF, whereas B9-2/GR cells were still incapable of even early morphologic maturation or substantial induction of *Mpo* RNA (Figure 6C, lower row and Figure 6D, bottom). Nearly complete terminal differentiation of B11-1 and B11-2 32Dcl3 cells despite reduction in *Cebpa* to an extent greater than the average 3-fold reduction seen in marrow cells transduced with *Cebpa* shRNAs suggests that impaired marrow granulopoiesis upon B9 or B11 transduction reflects lack of commitment to the granulocytic lineage rather than defective granulocytic maturation.

### C/Ebpα-ER Rescues the Effect of *Cebpa* Knockdown in Murine Marrow or 32Dcl3 Cells

**Figure 7:**
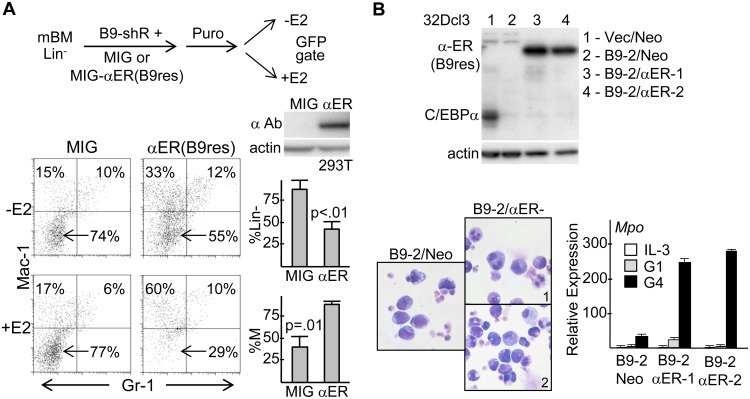
C/EBPα-ER rescues the differentiation blocked induced in murine marrow or 32Dcl3 cells by *Cebpa* knockdown. **A**) Diagram of marrow transduction protocol with lentiviral pLKO.1(puro)-B9 shRNA and MIG or MIG-C/EBPα-ER(B9res) retrovirus (top). 293T whole cell extracts obtained 2 days after transfection with MIG or MIG-C/EBPα-ER(B9res) DNAs were subjected to Western blotting with C/EBPα or actin antibodies (center, right). Representative Mac-1;Gr-1 FACS analysis of marrow cells 2 days after transfer to IL-3, IL-6, SCF +/− E2 (bottom, left). Average reduction in Mac-1^−^Gr-1^−^(Lin^−^) cells or increase in percent monocytes compared to total monocytes + granulocytes (n = 3, bottom, right). **B**) Two subclones of B9-2 cells transduced with pBabeNeo-C/EBPα-ER(B9res), B9-2/Neo and Vec/Neo cells were subjected to Western blotting for C/EBPα and β-actin (left). B9-2/Neo, B9-2/αER-1, and B9-2/αER-2 cells cultured in G-CSF with estradiol were subjected to Wright-Giemsa staining on G6, the day of maximal differentiation (center). RNAs prepared in IL-3 or G-CSF with simultaneous addition of estradiol were analyzed for *Mpo* expression (right, n = 3).

Besides use of two shRNAs, as a further control for shRNA off-target effects, we developed a variant of C/EBPα-ER rendered resistant to B9 shRNA via synonymous codon mutations. Marrow cells were simultaneously transduced with pLKO.1(puro)-B9 shRNA and MIG or MIG-C/EBPα-ER(B9res) vectors, puromycin selected, transferred to IL-3, IL-6, and SCF +/− estradiol (E2), and subjected to FACS, gating on GFP^+^ cells (Figure 7A). Western blotting of transfected 293T cells confirmed expression of C/EBPα-ER(B9res). The proportion of immature Mac-1^−^Gr-1^−^ cells that accumulate with B9 shRNA was reduced more than 2-fold by activation of C/EBPα-ER(B9res), indicating induction of their myeloid differentiation. There was some reduction even in the absence of estradiol likely due to ER leakiness. There was also an increase in the ratio of Mac-1^+^Gr-1^−^ to Mac-1^+^Gr-1^+^ cells, as we had seen previously with expression of C/EBPα-ER in wild-type murine marrow cells, potentially reflecting formation of C/EBPα-ER:AP-1 heterodimers [8], [17].

32Dcl3(B9-2) cells were transduced with shRNA-resistant pBabeNeo-C/EBPα-ER(B9res), and two subclones demonstrating transgene expression were identified (Figure 7B, top). Addition of estradiol to these lines rescued full granulocytic differentiation in G-CSF (Figures 7B, bottom). Notably, the large majority of B9-2/αER cells died by day 2 in G-CSF in the absence of estradiol whereas there numbers increased 1.5- to 2-fold and then stabilized for 6 days in the presence of estradiol.

### Global RNA Expression Analysis of *Cebpa* Knockdown Lineage-negative Marrow Cells Reveals Known and Potentially Novel C/Ebpα Targets

**Figure 8:**
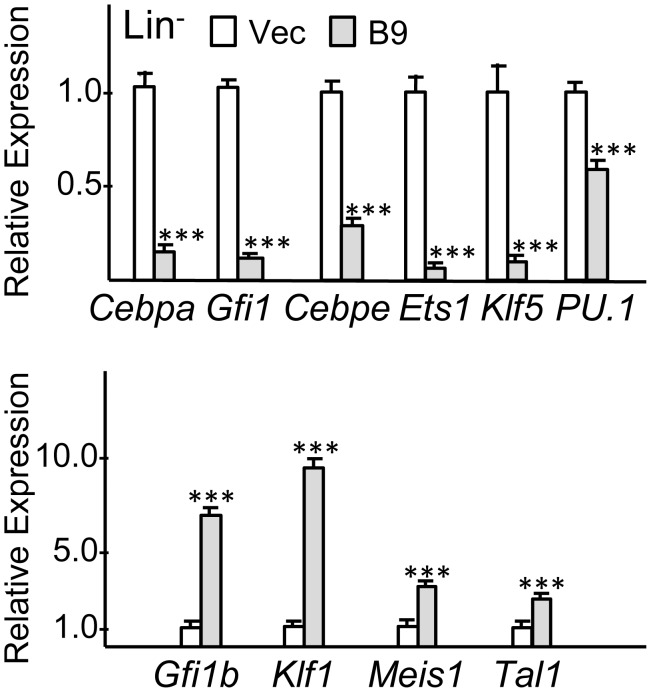
Quantitative RT-PCR analyses from RNA samples used for global gene expression analysis. RNA samples from replicate pLKO.1 and B9 transductions were subjected to quantitative RT-PCR in triplicate for indicated mRNAs relative to β-actin RNA. Expression in pLKO.1 vector samples was set to 1.0 on average in each experiment, and mean Relative Expression values in the two B9 samples are shown.

Murine marrow was transduced with the pLKO.1 or B9 shRNA for 2 days in TPO/FL/SCF, puromycin-selected in these same cytokines for 2 additional days, then cultured for 2 days in IL-3/IL-6/SCF, and finally lineage-depleted using Mac-1, Gr-1, Ter119, B220, and CD3 antibodies. mRNAs were then subjected to gene expression analysis using Illumina arrays. Biologic replicate samples were analyzed from independent transductions. A list of genes increased or decreased at least 1.4-fold in both experiments was obtained (Table S2). A subset of these genes, representing lineage markers and regulatory proteins, was also compiled (Table 1). For RNAs expressed near background the entries in these tables underestimate the true fold-change. The same RNA samples were subjected to RT-PCR to validate several of these results (Figure 8). Each of 6 RNAs decreased and 4 RNAs increased >1.4-fold in the array were indeed decreased or increased at least to this extent. *Cebpa* RNA was decreased 6-fold by B9 shRNA, on average; *PU.1* was decreased 1.6-fold. In comparable lineage-negative cells, B11 shRNA reduced *Cebpa* 1.4-fold (not shown).

**Table 1:** Selected mRNAs decreased or increased in Lin^−^ marrow cells by *Cebpa* shRNA*.

Gene name	Fold-change	Descriptive name	Gene name	Fold-change	Descriptive Name
*Aatk*	−2.8/2.2	Apoptosis-associated tyr kinase	*Mapk31*	−1.9/1.7	MAP kinase 11 (MLK3)
*Camp*	−7.7/8.3	Cathelicidin antimicrobialpeptide	*Map3k3*	−1.9/1.6	MAP kinase kinase 3
*Cebpa*	−1.9/1.8	C/EBPα transcriptionfactor	*Med21*	−2.1/1.6	Mediator complexsubunit 21
*Cebpe*	−4.4/3.4	C/EBPε transcriptionfactor	*Mpo*	−2.2/1.8	Myeloperoxidase
*Csf3r*	−1.5/1.6	G-CSF receptor	*Nfe2l2*	−1.5/1.4	NF-E2 like 2
*Csf2ra*	−1.5/1.7	GM-CSF receptor α subunit	*Nras*	−2.3/1.8	N-Ras
*Ctsb*	−1.6/1.6	Cathepsin B	*Prg2*	−4.3/3.6	Eosinophil majorbasic protein
*Ctsg*	−1.9/1.7	Cathepsin G	*Prkcb*	−1.9/1.6	Protein kinase C β
*Ctsgh*	−1.9/1.8	Cathepsin H	*Prss34*	−47/−19	Mast cell tryptase 34
*Cxcr4*	−1.7/1.6	Cxcr4 chemokinereceptor	*Prtn3*	−2.7/2.2	Proteinase 3
*Dgkg*	−2.0/2.0	DAG kinase γ	*Rab27a*	−2.6/2.2	Rab27a GTPase
*Dnmt31*	−2.2/2.4	DNA-methyltransferase3-like	*Rab32*	−1.8/2.0	Rab32 GTPase
*Ela2*	−10/5.6	Neutrophil elastase	Sfpi1**	−1.6/1.3	PU.1 transcriptionfactor
*Epx*	−3.2/2.5	Eosinophil peroxidase	*Tcn2*	−2.3/2.2	Trans-cobalamin 2
*Ets1*	−3.0/1.8	Ets1 transcription factor	*Alad*	+2.0/2.1	Aminolevulinae dehdratase
*Fcgr2b*	−2.0/1.6	Fcγ receptor 2b	*Bcl11*	+1.8/1.7	Bcl11A transcription factor
*Fcgr3*	−3.2/2.2	Fcγ receptor 3	*Ccnd1*	+5.0/3.6	Cyclin D1
*Fkbp11*	−2.5/1.7	Peptidyl prolineisomerase	Cd14**	+1.7/1.3	CD14
*Gadd45a*	−1.9/1.8	Growth arrest/DNA-damage 45a	*Cd34*	+1.8/1.7	CD34
*Gapt*	−3.8/2.9	GRB2-binding adaptorprotein	*Cd52*	+4.1/2.7	CD52
*Gfi1*	−5.0/3.7	Gfi-1 transcriptionfactor	*Dnmt3b*	+2.5/2.0	DNA-methyltransferase 3b
*Hgf*	−2.5/1.5	Hepatocyte growthfactor	*Gfi1b*	+3.7/3.6	Gfi-1b transcriptionfactor
*Idh1*	−1.7/1.8	Isocitratedehydrogenase I	*Hba-a1*	+22/16	Hemoglobin α
*Ifngr1*	−1.8/1.9	Interferon γreceptor 1	*Hba-b1*	+5.2/5.4	Hemoglobin β
*Ikbe1*	−1.7/1.5	IκB kinase ε	*Klf1*	+2.6/2.3	Klf1 transcriptionfactor
*Irak3*	−1.9/1.5	IL-1 receptorassociated kinase	*Meis1*	+1.7/1.7	Meis1 transcriptionfactor
*Klf5*	−2.2/1.4	Klf5 transcriptionfactor	*Nfkbia*	+1.5/1.4	IκBα
*Lrb4r1*	−4.2/3.2	Leukotriene B4receptor	*Pip4k2a*	+1.9/1.6	PI-5-phosphate-4-kinase
*Ltf*	−3.1/2.0	Lactoferrin	*Tal1*	+1.8/2.2	Tal1/SCL transcriptionfactor
*Lyz*	−1.5/2.0	Lysozyme	*Tnf*	+1.6/1.6	Tumor necrosis factor

*Fold-change at least 1.4-fold, except as marked by **, in two transductions with vector and B9-shRNA.

(−) decreased, (+) increased by shRNA B9.

Granulocytic markers decreased by B9 in the lineage-negative population included *Csf3r*, *Ela2*, *Ltf*, *Mpo*, *Prtn3*, and *Tcn2*, whereas, monocytic markers *Cd14* and *Tnf* were increased. Cathepsins, Fcγ receptor, and lysozyme, expressed by both lineages, were also reduced. *Cebpe* and *Gfi1*, known transcriptional regulators of granulopoiesis, were reduced, as were additional transcription factors, *Ets1*, *Klf5*, and *Nfe2l2*. *Klf4* and *Irf8* were not altered substantially on the array, and RT-PCR analysis revealed little change in *Cebpb*, *JunB*, or *c-Fos* and only 1.6-fold increase in *c-Jun* (not shown). *Ikbe* was decreased and *Nfkbia* was increased by *Cebpa* knockdown, suggesting a normal role for C/EBPα in up-regulating NF-κB signaling. Ingenuity pathway analysis (Table 2) identified several additional pro-inflammatory pathways reduced by *Cebpa* knockdown, including those mediated by IL-8, IL-10, iNOS, or LPS signaling. The specific genes altered in these pathways are also listed.

**Table 2:** Top pathways with affected genes altered by *Cebpa* knockdown*.

Pathway	−logP	R	Genes
Production of NOand reactive O_2_ inmacrophages	4.74	0.076	*MAP3K11,MAP3K6,MPO,IKBKE,IFNGR1,TLR4,NFKBIA,* *RHOB, RND3,CYBA,RHOU,S100A8,TNF,MAP3K3,SIRPA,PRKCB*
Interleukin-10signaling	3.43	0.103	*NFKBIA,FCGR2A,BLVRB,IL1B,IKBKE,LBP,* *FCGR2B,TNF*
LPS/IL-1mediatedinhibition of RXRfunction	3.20	0.063	*ALDH4A1,MGST1,GSTM5,CHST12,PAPSS2,TLR4,HS6ST1*,*MGST2, IL1B, ALAS1,ALDH3B1,LBP,CHST13,TNF,ACSL1*
Inhibitor of NOsynthase (iNOS)signaling	3.04	0.113	*TLR4,NFKBIA,IKBKE,IFNGR1,LBP,IRAK3*
Aryl hydrocarbonreceptor signaling	2.99	0.068	*TGM2,ALDH4A1,MGST1,MGST2,GSTM5,CDKN1A,IL1B*,*ALDH3B1, CCND1,TNF,NFE2L2*
Interleukin-8signaling	2.97	0.064	*VCAM1,NRAS,ANGPT1,MPO,IKBKE,IRAK3,CCND1,* *PLD1,CSTB, RND3,RHOB,RHOU,PRKCB*
Hepatocytegrowth factorsignaling	2.96	0.086	*ETS1,NRAS,MAP3K11,MAP3K6,HGF,CDKN1A,CCND1,* *MAP3K3,PRKCB*
CD27 signaling inlymphocytes	2.70	0.105	*NFKBIA,MAP3K11,MAP3K6,IKBKE,* *CD27,MAP3K3*
B cell receptorsignaling	2.66	0.067	*ETS1,NRAS,NFKBIA,MAP3K11,MAP3K6,FCGR2A,PIK3AP1,IKB* *KE, FCGR2B,MAP3K3,PRKCB*
High mobilitygroup box 1(HMGB1)signaling	2.54	0.081	*TLR4,VCAM1,NRAS,RND3,RHOB,RHOU,IFNGR1,TNF*
Xenobioticmetabolismsignaling	2.48	0.054	*ALDH4A1,MGST1,MAP3K11,NRAS,MAP3K6,GSTM5,CHST12,* *HS6ST1,MGST2,IL1B,ALDH3B1,CHST13,NFE2L2,TNF,MAP3K3,* *PRKCB*
Cholinebiosynthesis	2.39	0.136	*PCYT1B,CHPT1,PLD1*
Vitamin DReceptor/RXRactivation	2.38	0.086	*SERPINB1,SPP1,CAMP,GADD45A,CDKN1A,* *CEBPA,PRKCB*
TREM1 signaling	2.38	0.085	*TLR4,LAT2,MPO,IL1B,FCGR2B,TNF*
TNFR2 signaling	2.25	0.121	*TANK,NFKBIA,IKBKE,TNF*
PI-3-kinasesignaling in Blymphocytes	2.20	0.064	*TLR4,NRAS,C3,NFKBIA,ATF5,PIK3AP1,* *IKBKE,FCGR2B,PRKCB*

*Top pathways from analysis of RNAs with >1.5-fold change in 2 experiments, with affected genes listed and ordered by –logP values. R values, indicating the proportion of genes in a pathway affected by *Cebpa* knockdown, are also shown.

Unexpectedly, several lineage markers and regulators of erythropoiesis were increased by *Cebpa* knockdown, including α-globin, β-globin, aminolevulinate dehydrogenase, and transcriptional regulators *Bcl11A*, *Gfi1b*, *Klf1*, and *Tal1*, the latter three confirmed by RT-PCR analysis (Figure 8). Given these findings and increased MEP seen by FACS, we compared erythroid colony formation from pLKO.1 versus B9-transduced marrow cells in methylcellulose with EPO. *Cebpa* knockdown increased Ter119^+^ BFU-E numbers (Figure S3), with hemoglobinization evident in cell pellets (not shown).

### *Cebpa* RNA Is Elevated in a c-Kit^+^GCSFR^+^ Population Enriched for CFU-G Compared to a c-Kit^+^MCSFR^+^ Population Enriched for CFU-M

**Figure 9:**
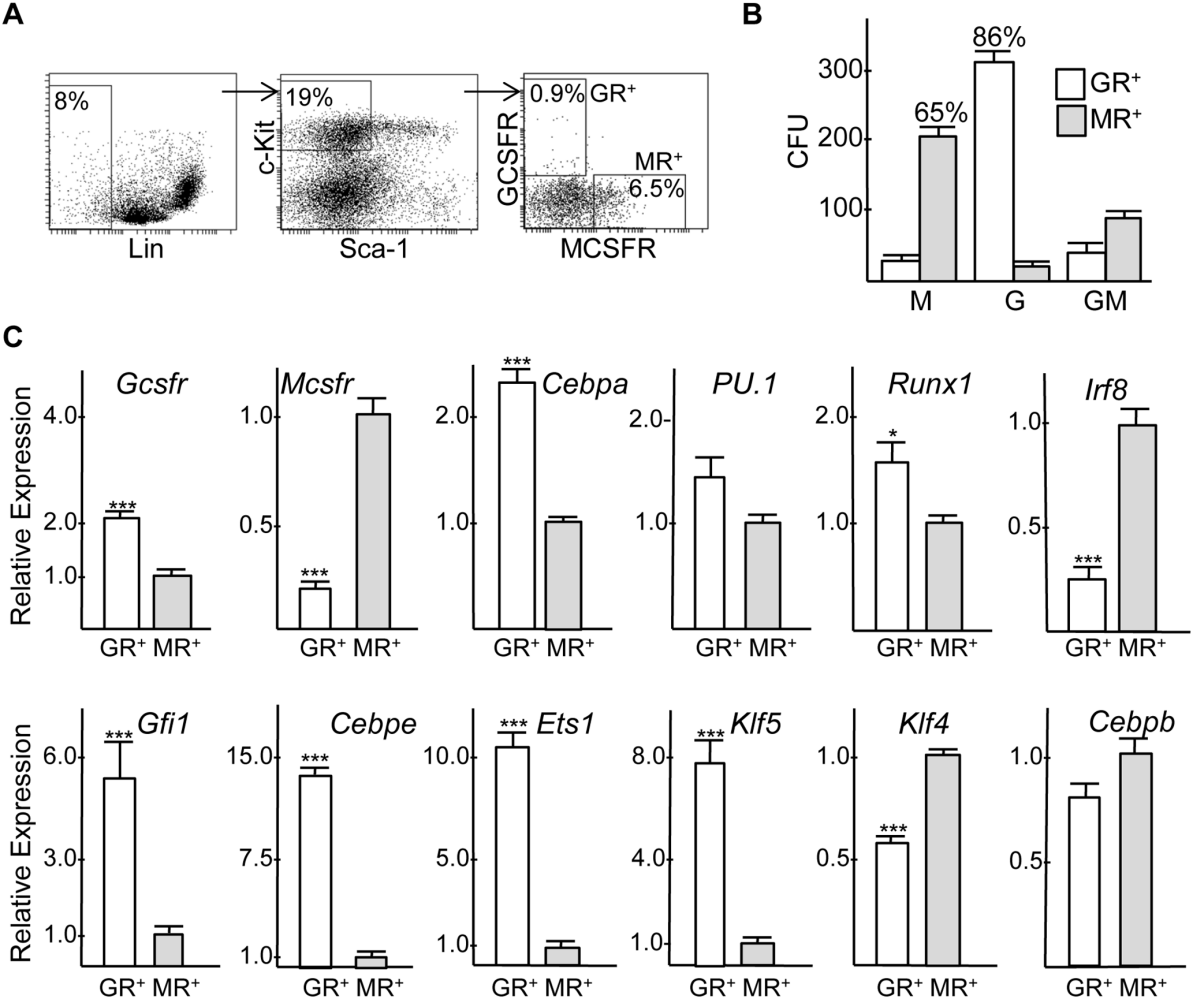
Myeloid CFUs and RNAs expression in Lin^−^Sca-1^−^c-Kit^+^ murine marrow cells expressing G-CSF versus M-CSF receptor. **A**) FACS analyses demonstrating isolation of Lin^−^Sca-1^−^c-Kit^+^ GCSFR(GR)^+^MCSFR(MR)^−^ and Lin^−^Sca-1^−^c-Kit^+^GCSFR(GR)^−^MCSFR(MR)^+^ populations. **B**) The number of CFU-M, CFU-G, or CFU-GM obtained per 1E4 cells plated in methylcellulose culture with IL-3, IL-6, and SCF is shown (n = 3). **C**) Total cellular RNAs were subjected to quantitative RT-PCR for indicated mRNAs (n = 3).

Murine marrow cells were stained for Lineage markers, c-Kit, Sca-1, GCSFR, and MCSFR (Figure 9A). The immature Lin^−^ Sca-1^−^c-Kit^+^ subset was sorted into GCSFR^+^MCSFR^−^ (GR^+^MR^−^) and GCSFR^−^MCSFR^+^ (GR^−^MR^+^) subsets followed by plating in methylcellulose with IL-3, IL-6, and SCF (Figure 9B). On average, 86% of the CFUs obtained from the GR^+^MR^−^ population were CFU-G, while 65% of the CFUs obtained from the GR^−^MR^+^ population were CFU-M. The majority of the remaining CFUs were CFU-GM. CFUs represented 11% of sorted GR^+^MR^−^ and 9% of GR^−^MR^+^ cells, indicated that the remainder where either progenitors that do not form colonies under these culture conditions or are immature precursors. Under the same culture conditions, 15% of sorted GMP cells formed myeloid CFUs (not shown). Quantitative RNA analysis from three independent experiments confirmed enhanced expression of *Gcsfr* in the c-Kit^+^GR^+^MR^−^ and *Mcsfr* in the c-Kit^+^GR^−^MR^+^ populations and demonstrated that *Cebpa* RNA is expressed on average 2.3-fold higher in the myeloid progenitor population enriched for CFU-G compared to that enriched for CFU-M (Figure 9C). *PU.1* and *Runx1* were mildly enriched in the GR^+^MR^−^ cells. *Cebpe*, *Gfil1*, *Ets1*, and *Klf5* RNAs were expressed at markedly higher levels in the CFU-G-enriched population, whereas *Klf4* and *Irf8* were increased in the CFU-M-enriched subset.

## Discussion

Deletion of the *Cebpa* alleles in adult marrow leads to marked reduction in GMP, making the subsequent role of C/EBPα in granulocyte versus monocyte lineage specification uncertain. Providing new insight into the role of C/EBPα in myeloid lineage specification, we now demonstrate that partial *Cebpa* knockdown impairs granulopoiesis while increasing monopoiesis with further reduction blocking commitment to either myeloid lineage. The shRNA producing the most striking phenotypic alteration reduced *Cebpa* 3-fold in the total population of differentiating myeloid cells and 6-fold in accumulating blasts. Blast expansion was transient *in vivo*, indicating that further genetic alterations would be required to avoid quiescence or cell death to obtain indefinite expansion and perhaps malignant transformation. As controls for off target effects, a shift from granulopoiesis toward monopoiesis was obtained with two different *Cebpa* shRNAs in murine marrow and with two different *CEBPA* shRNAs in human CD34^+^ cells, and shRNA-resistant C/EBPα-ER overcame the effects of *Cebpa* knockdown.

*Cebpa* knockdown reduced CFU-G while increasing CFU-M. Increased CFU-M suggests a shift in lineage commitment rather than simply a defect in granulocytic maturation – in support of this conclusion, *Cebpa* knockdown in 32Dcl3 cells to an extent greater than that obtained in total marrow cells did not prevent their near terminal maturation in response to G-CSF. Thus, higher levels of C/EBPα are apparently needed for granulocyte compared to monocyte lineage specification. Accumulation of immature blasts in response to *Cebpa* knockdown suggests that low level C/EBPα expression is still required for monopoiesis, as these blasts may represent myeloid progenitors that express C/EBPα below a requisite threshold. Of note, although exogenous C/EBPα blocks G1 to S cell cycle progression in myeloid and non-myeloid cells [8], [18], *Cebpa* knockdown did not alter cell cycle parameters in transduced marrow cells. Also, reduced *Cebpa* led to increased CFU replating but not to cytokine-independence.

C/EBPα expression or activity is reduced by multiple mechanisms in acute myeloid leukemia (AML) [19]. Accumulation Lin^−^Sca-1^+^c-kit^+^ stem cells *in vitro* and *in vivo* and extensive colony replating upon *Cebpa* knockdown may represent a preleukemic state, with additional mutations required for full, cytokine-independent transformation. Human AMLs or murine marrow completely lacking C/EBPα have increased C/EBPγ [20]; however, *Cebpg* was not increased in Lin^−^ marrow or 32Dcl3 lines expressing B9 or B11 *Cebpa* shRNA (Figure S4). Of note, *RUNX1* is commonly mutated in AML, and *Runx1* gene deletion in adult mice reduces *Cebpa* mRNA and increases *in vitro* monopoiesis while reducing granulopoiesis, similar to the effect of *Cebpa* knockdown [12]. The present study indicates that *Cebpa* may be the *Runx1* target gene most critical for the impaired granulopoiesis and increased myeloproliferation evident in the absence of *Runx1*.

In contrast to our findings with *Cebpa* knockdown, reduced PU.1 expression favors granulopoiesis over monopoiesis [21]–[23]. C/EBPα activates the *PU.1* promoter and −14 kb distal enhancer [24], [25], and herein *Cebpa* knockdown reduced *PU.1* mRNA 1.6-fold, to about 60% of control levels, in lineage-negative marrow cells. Yet, monopoiesis was favored by *Cebpa* shRNA transduction, potentially reflecting the greater decrease in *Cebpa* RNA. A similar explanation may account for our finding that *Runx1* gene deletion mimics *Cebpa* knockdown. Runx1 activates *Cebpa* gene expression via binding sites in the *Cebpa* promoter and via a +37 kb enhancer and activates *PU.1* transcription via the −14 kb distal enhancer [13], [26]; however, *Runx1* gene deletion impairs *Cebpa* RNA expression to a greater extent than *PU.1* in CMP and GMP [12].

Our results predict that granulocytic progenitors have either increased C/EBPα expression or similar expression but increased C/EBPα activity, due to protein modifications or interactions, compared with monocytic progenitors. As a first step to evaluate these possibilities we developed a FACS protocol sorting Lin^−^ Sca-1^−^c-Kit^+^ cells based on GCSFR versus MCSFR expression that allows enrichment for immature granulocytic versus monocytic progenitors/precursors. Although these sorted populations were not pure CFU-G or CFU-M, the increased *Cebpa* RNA seen in the GR^+^MR^−^ compared with the GR^−^MR^+^ population suggests that *Cebpa* RNA is elevated in CFU-G and immature precursors they generate compared with CFU-M and their immediate progeny. Of note, the GR^−^MR^+^ population contains more CFU-GM than the GR^+^MR^−^ population, eliminating CFU-GM as the source of increased *Cebpa* RNA in the GR^+^MR^−^ subset. While future efforts might uncover a means to obtain pure, CFU-G or CFU-M, our approach is the first to reveal insight into changes in gene expression between early granulocyte versus monocyte lineage-committed cells.

What pathways might direct increased *Cebpa* expression to granulocyte progenitors? As discussed, Runx1 activates the *Cebpa* promoter and +37 kb enhancer. *Runx1* is elevated 1.6-fold in the GR^+^MR^−^ compared with the GR^−^MR^+^ population. In addition, Runx1 might be more active in the granulocytic lineage: G-CSF activates the SHP2 tyrosine phosphatase more potently than M-CSF, SHP2 knockdown reduces *Cebpa* but not *PU.1* to impair granulopoiesis relative to monopoiesis [12], [27], and SHP2 may directly activate Runx1, at least in the megakaryocyte lineage [28]. Also of potential relevance, SHP2 also activates Src kinases in hematopoietic cells [29], and Src-mediated tyrosine phosphorylation of Runx1 increases its activity in 293T cells and favors granulopoiesis in *Runx1*-deleted marrow cells (our unpublished observations).

Elevated levels of C/EBPα might allow formation of C/EBPα:C/EBPα homodimers capable of activating targets such as *Gfi1* and *Cebpe* required for granulopoiesis. In monocytic progenitors, reduced levels of C/EBPα may instead form C/EBPα:AP-1 heterodimers, mediated by their leucine zipper domains. Of note, dimerization of C/EBPα with AP-1 proteins stimulates murine marrow monopoiesis, and C/EBP:AP-1 hybrid elements are enriched in promoter and enhancer regions of multiple monocyte-specific genes [17], [30], [31]. M-CSF activates ERK more potently than G-CSF, ERK inactivates C/EBPα via serine phosphorylation, and chemical ERK inhibition favors granulopoiesis [27], [32]. Thus, not only *Cebpa* expression but also C/EBPα activity might be regulated during myeloid lineage specification, with inactive C/EBPα still capable of forming active complexes with AP-1 proteins to help direct monopoiesis.

Consistent with prior studies showing that the murine *Cebpa*(−/−) fetal liver has increased BFU-E and that exogenous C/EBPα inhibits erythropoiesis in transduced human CD34^+^ marrow cells [33], [34], *Cebpa* knockdown increased MEP, BFU-E, and expression of erythroid-specific genes in lineage-negative marrow progenitors. This effect may in part reflect inhibition of *PU.1* expression, as PU.1 directly binds GATA-1 to inhibit erythropoiesis [9], [10]. On the other hand, the modest reduction in *PU.1* RNA in response to *Cebpa* knockdown raises the possibility that C/EBPα impedes erythropoiesis through additional mechanisms as well.

Global gene expression analysis of lineage-negative marrow progenitors after vector or *Cebpa* shRNA transduction and culture in myeloid cytokines confirmed reduction in *Gfi1* and *Cebpe,* known regulators of granulopoiesis and direct targets of C/EBPα [5], [35], and also demonstrated a decrease in *Ets1* and *Klf5.* Strikingly correlating with these findings, GR^+^MR^−^ cells were not only enriched for *Cebpa* but also for *Gfi1*, *Cebpa*, *Ets1*, and *Klf5*, underscoring their potential importance as C/EBPα-regulated targets relevant to granulopoiesis. Of note, exogenous C/EBPα induces *Klf5* in human CD34^+^ marrow cells, *Klf5* is increased in granulocytes compared with monocytes or erythroblasts, *Klf5* knockdown impairs 32Dcl3 or NB4 granulopoiesis, and *Klf5* deletion reduces murine myelopoiesis [34], [36]–[38]. Interestingly, *Klf4* antagonizes *Klf5* in non-hematopoietic lineages, exogenous *Klf4* directs marrow monopoiesis, and we find *Klf4* RNA enriched in the GR^−^MR^+^ population [39].

Given substantial data, discussed above, indicated that increased PU.1 favors monopoiesis over granulopoiesis, it is also noteworthy that PU.1 levels were similar in the GR^+^MR^−^ and GR^−^MR^+^ subsets. In contrast, *Irf8* was markedly elevated in the GR^−^MR^+^ population. Irf8 forms a complex with PU.1 to direct binding to composite Ets/IRF DNA elements, and Irf8 is required for monopoiesis [40], [41]. Thus, graded PU.1 activity, rather than expression, in the two myeloid lineages might be achieved via graded *Irf8* expression.

*Cebpa* knockdown reduced *Ikbke* and increased *Nfkbia*, thereby predicting impaired NF-κB activation, and indeed pathway analysis uncovered suppression of several pro-inflammatory pathways. Mice lacking NF-κB p50 have 3-fold reduced C/EBPα RNA and protein, 2-fold reduced CFU-G, and mildly increased CFU-M [42], suggesting that impairment of NF-κB activation through changes in NF-κB p50, IκB kinase ε, and IκBα expression may contribute to the reduced granulopoiesis observed with *Cebpa* knockdown.

## Supporting Information

Figure S1 **Myeloid colony morphologies. A**) Typical murine CFU-G, CFU-M, and CFU-GM. **B**) Typical human CFU-G, CFU-M, and CFU-GM. Images were obtained at 40X using a Nikon Eclipse Ti microscope. All six CFUs are shown at the same scale.
(TIF)

Figure S2 ***Cebpa*** **knockdown increases monocyte relative to granulocyte formation in comparison with empty (Vec) or non-targeting vector (NTV) lentiviral controls.** Transduced, puromycin selected marrow cells were placed in liquid culture with IL-3, IL-6, and SCF. FACS analyses for Mac-1 and Gr-1 on D2 are shown. Arrows indicate immature, Mac-1^−^Gr-1^−^ cells.
(TIF)

Figure S3 ***Cebpa*** **knockdown increases erythroid colonies. A**) Marrow cells transduced with the pLKO.1 vector or with *Cebpa* shRNA B9 were selected in puromycin and then plated in methylcellulose with EPO at 1.6E5 cells/ml, and BFU-E were enumerated 7 days later (n = 3). **B**) Pooled CFU cells were subjected to FACS analysis for Ter119.
(TIF)

Figure S4 **High-level** ***Cebpa*** **knockdown does not reduce** ***Cebpg*** **in murine marrow progenitors or in the 32Dcl3 cell line. A**) RNA samples from replicate pLKO.1 (Vec) and B9 transductions were subjected to quantitative RT-PCR in triplicate for *Cebpg* relative to β-actin RNA. Expression in pLKO.1 vector samples was set to 1.0 on average in each experiment, and mean Relative Expression values in B9 samples are shown. **B**) Similar analysis was conducted for RNAs prepared from parental 32Dcl3 cells or the 32Dcl3 pLKO.1, B11-1, or B9-2 lines. Average expression in the vector line was set to 1.0.
(TIF)

Table S1 **RT-PCR primer pairs.**
(PDF)

Table S2 **Fold-change (FC) of indicated RNAs by** ***Cebpa*** **B9 shRNA vs vector in lineage-negative marrow cells.**
(PDF)
