# Supplementary figures and images for: Granulopoiesis Requires Increased C/EBPα Compared to Monopoiesis, Correlated with Elevated Cebpa in Immature G-CSF Receptor versus M-CSF Receptor Expressing Cells
Source: PLoS One. 2014 Apr 21;9(4):e95784. doi: 10.1371/journal.pone.0095784 (PMC3994156; doi:10.1371/journal.pone.0095784)

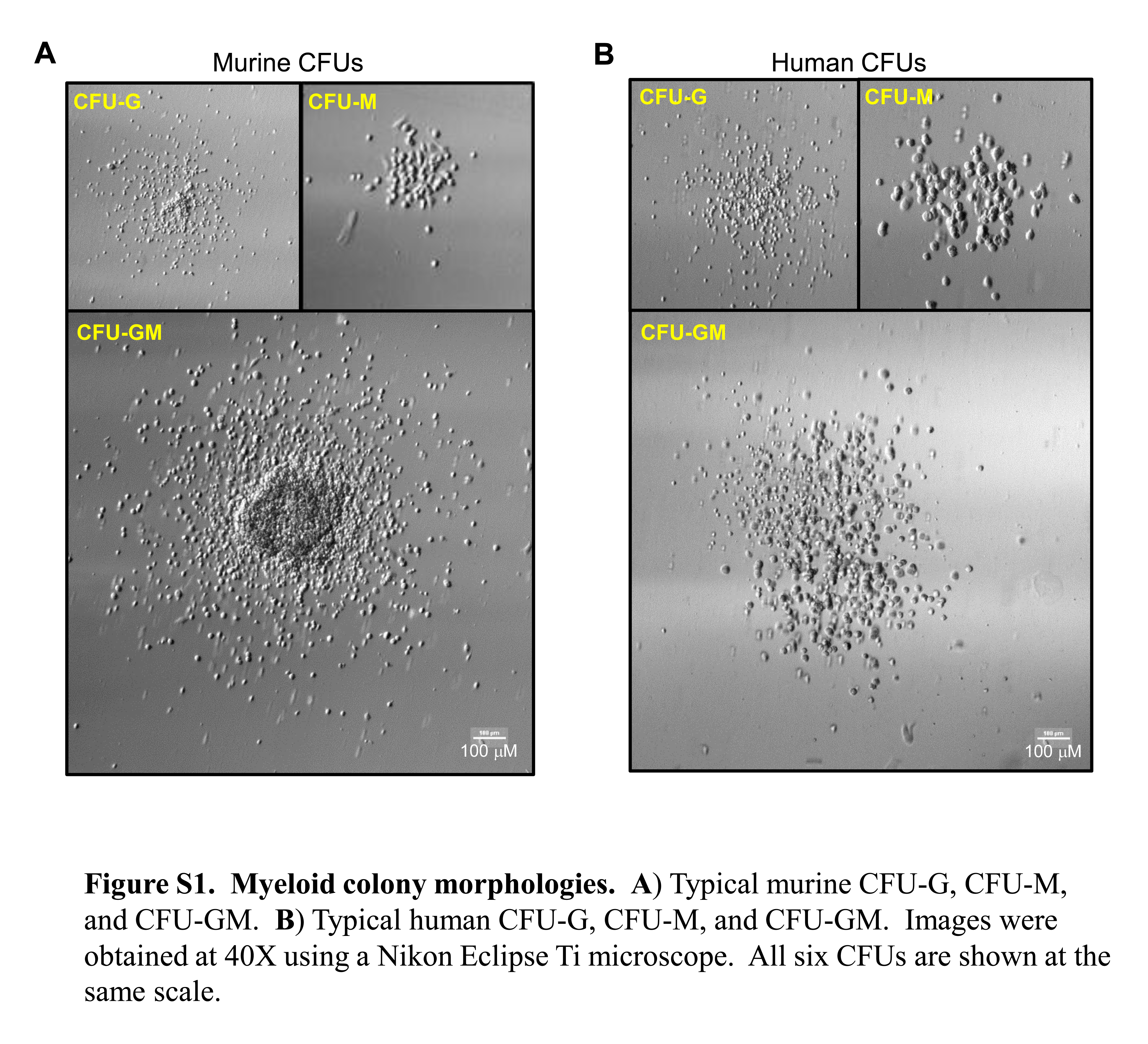

Supplement: Figure S1 — Myeloid colony morphologies. A) Typical murine CFU-G, CFU-M, and CFU-GM. B) Typical human CFU-G, CFU-M, and CFU-GM. Images were obtained at 40X using a Nikon Eclipse Ti microscope. All six CFUs are shown at the same scale. (TIF) [file pone.0095784.s001.tif]

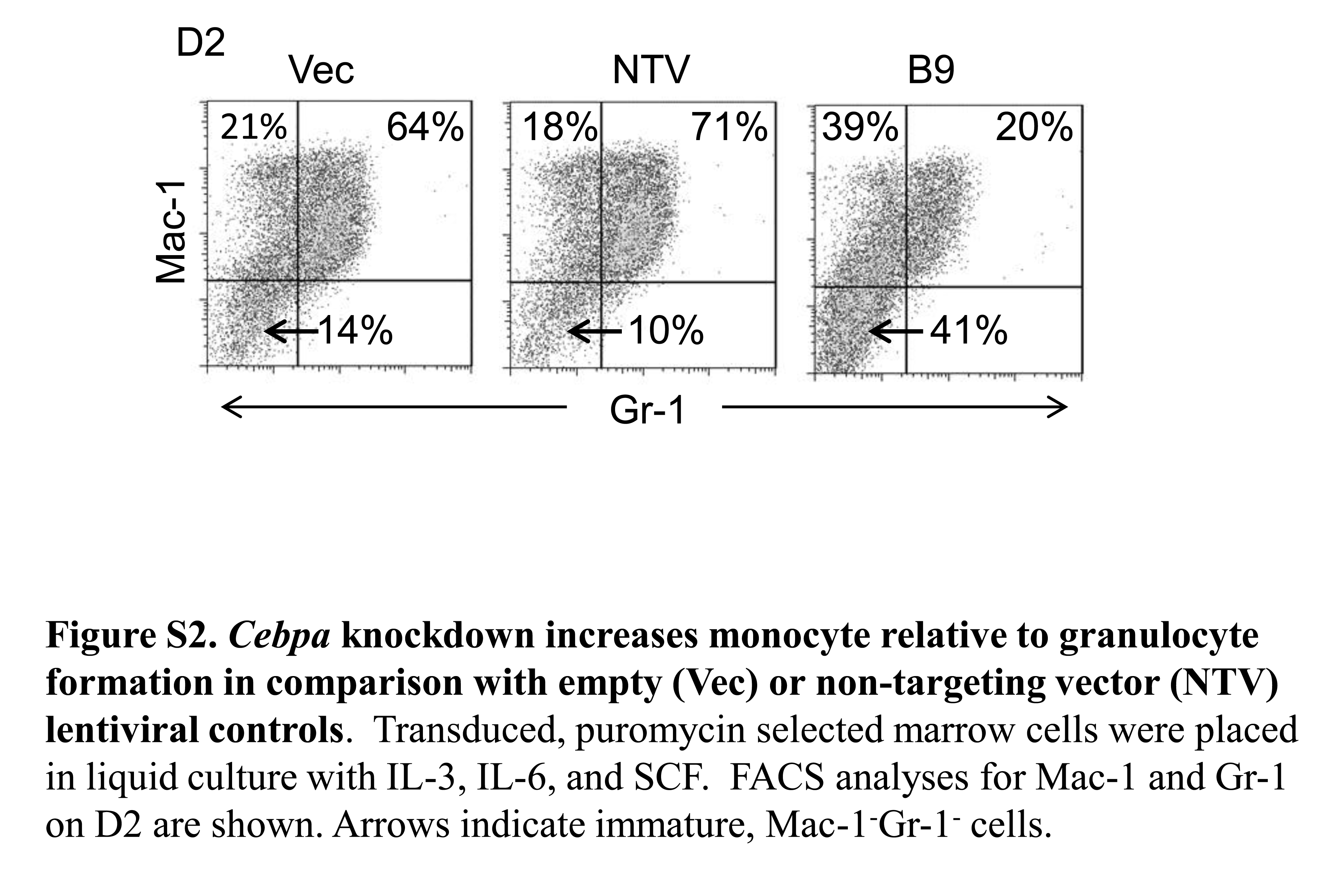

Supplement: Figure S2 — Cebpa knockdown increases monocyte relative to granulocyte formation in comparison with empty (Vec) or non-targeting vector (NTV) lentiviral controls. Transduced, puromycin selected marrow cells were placed in liquid culture with IL-3, IL-6, and SCF. FACS analyses for Mac-1 and Gr-1 on D2 are shown. Arrows indicate immature, Mac-1−Gr-1− cells. (TIF) [file pone.0095784.s002.tif]

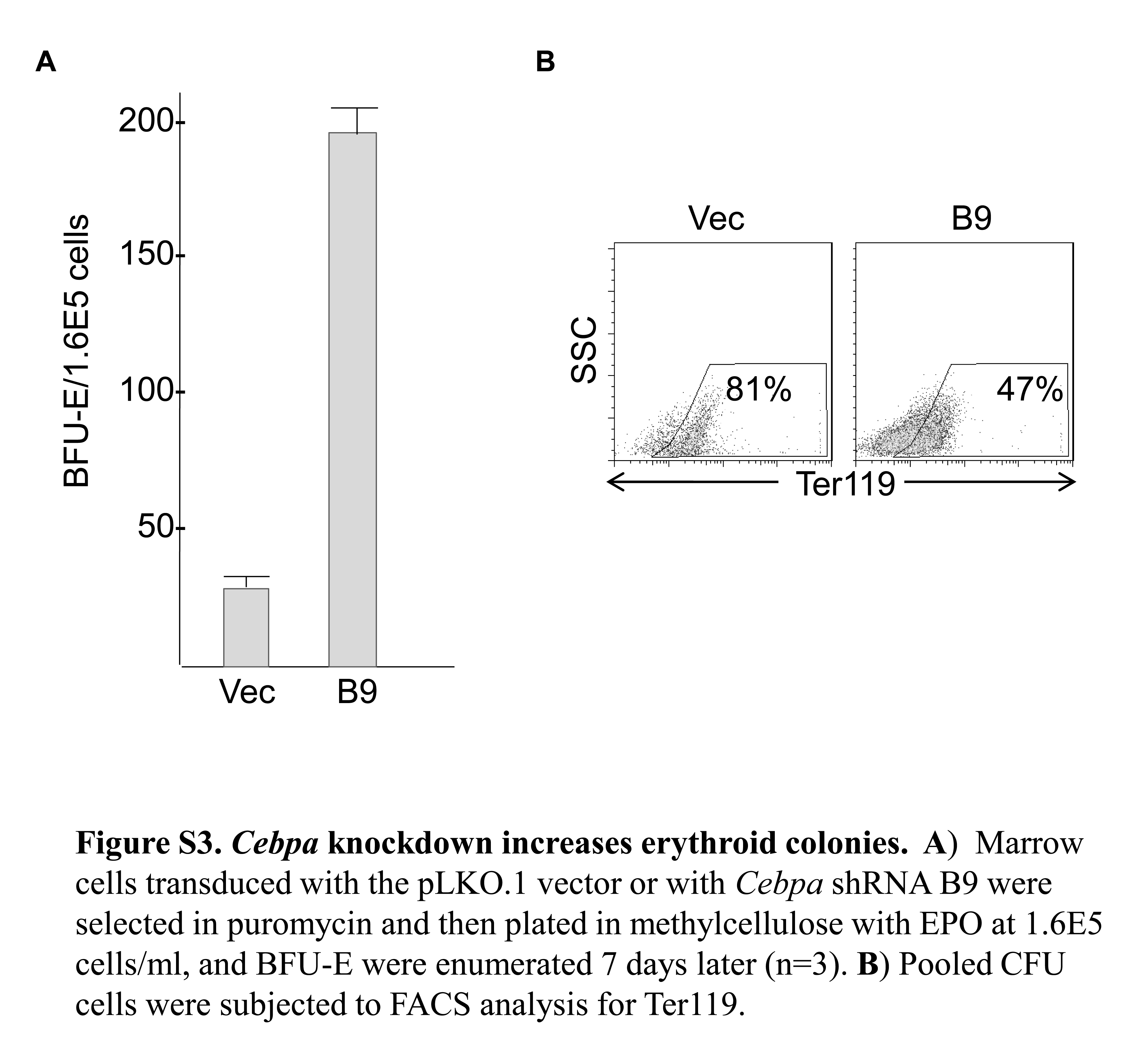

Supplement: Figure S3 — Cebpa knockdown increases erythroid colonies. A) Marrow cells transduced with the pLKO.1 vector or with Cebpa shRNA B9 were selected in puromycin and then plated in methylcellulose with EPO at 1.6E5 cells/ml, and BFU-E were enumerated 7 days later (n = 3). B) Pooled CFU cells were subjected to FACS analysis for Ter119. (TIF) [file pone.0095784.s003.tif]

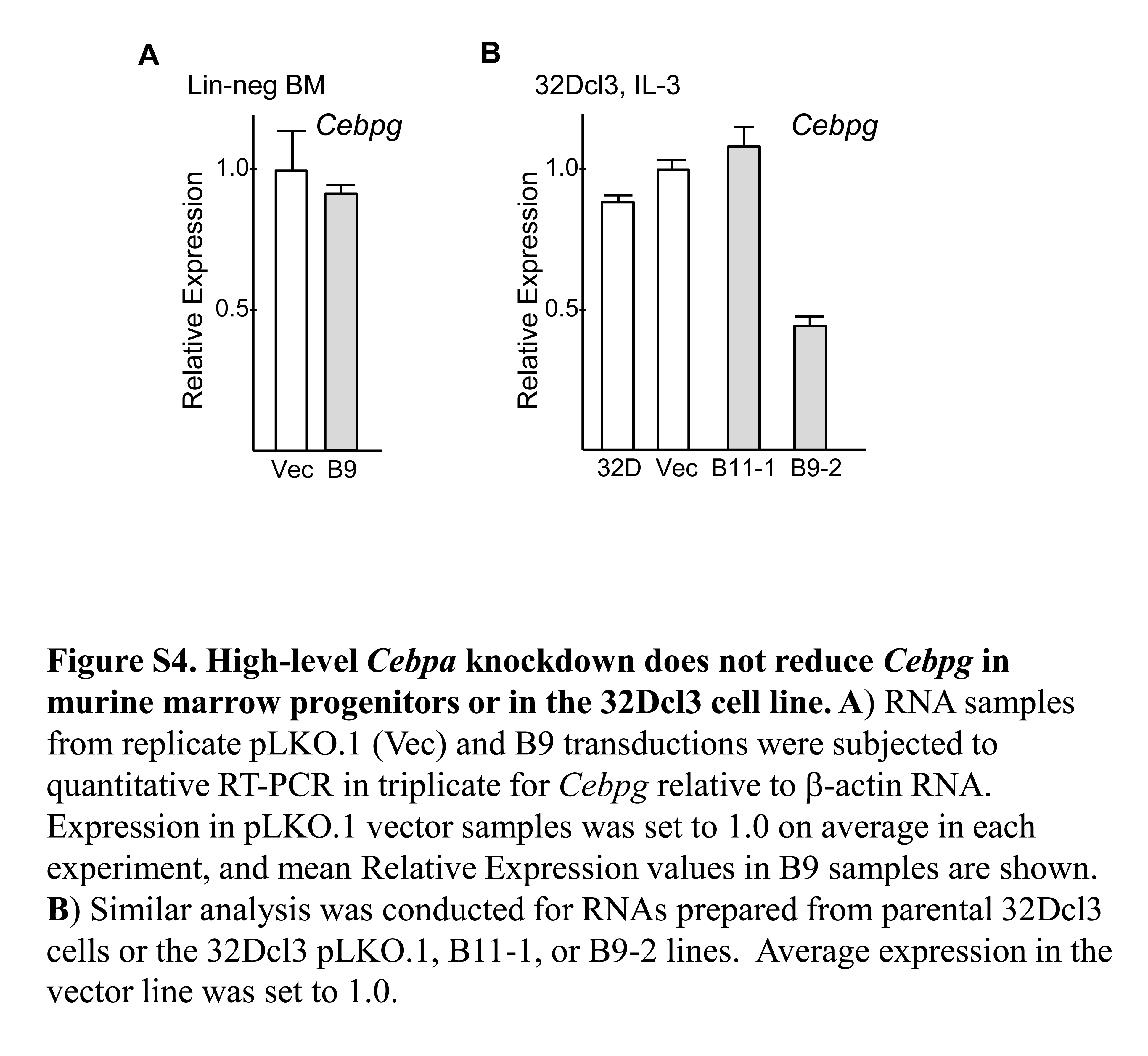

Supplement: Figure S4 — High-level Cebpa knockdown does not reduce Cebpg in murine marrow progenitors or in the 32Dcl3 cell line. A) RNA samples from replicate pLKO.1 (Vec) and B9 transductions were subjected to quantitative RT-PCR in triplicate for Cebpg relative to β-actin RNA. Expression in pLKO.1 vector samples was set to 1.0 on average in each experiment, and mean Relative Expression values in B9 samples are shown. B) Similar analysis was conducted for RNAs prepared from parental 32Dcl3 cells or the 32Dcl3 pLKO.1, B11-1, or B9-2 lines. Average expression in the vector line was set to 1.0. (TIF) [file pone.0095784.s004.tif]
