# Supplementary material for: Granulopoiesis Requires Increased C/EBPα Compared to Monopoiesis, Correlated with Elevated Cebpa in Immature G-CSF Receptor versus M-CSF Receptor Expressing Cells
Source: PLoS One. 2014 Apr 21;9(4):e95784. doi: 10.1371/journal.pone.0095784 (PMC3994156; doi:10.1371/journal.pone.0095784)
Supplement: Table S1 — RT-PCR primer pairs. (PDF) [file pone.0095784.s005.pdf]

**Table S1. RT-PCR primer pairs**

| | |
|---|---|
| *Bcl2* | F: ATGCCTTTGTGGAACTATATGGC<br>R: GGTATGCACCCAGAGTGATGC |
| *Cd14* | F: ATCTACCGACCATGGAGCGT<br>R: TCCACATCTGCCGCCCCCAA |
| *Cebpa* | F: CGGTGCGCAAGAGCCGAGAT<br>R: CCCGCAGCGTGTCCAGTTCA |
| *c-Myc* | F: GCTCTCCATCCTATGTTGCGG<br>R: TCCAAGTAACTCGGTCATCATCT |
| *Egr1* | F: GGCGATGGTGGAGACGAGT<br>R: CGGCCAGTATAGGTGATGGG |
| *Csf3r/Gcsfr* | F: CTGATCTTCTTGCTACTCCCCA<br>R: GGTGTAGTTCAAGTGAGGCAG |
| *Irf8* | F: AAGCAGGATTACAATCAGGAGGT<br>R: TCGGGGACAATTCGGTAAACT |
| *Csf1r/Mcsfr* | F: TCCGGTGGTGGTGGCCTGTA<br>R: AGCGCACCTGGTACTTCGGC |
| *Mpo* | F: GCTCCGCCCGCATTCCTTGT<br>R: TTGAGCTGTGTGGCCAGCCG |
| *Ela2/NE* | F: CAGGCATCTGCTTCGGGGAC<br>R: AGGGGCGAAGGCATCTGGGT |
| *Sfpi1/PU.1* | F: CCTTCGTGGGCAGCGATGGA<br>R: TGTAGCTGCGGGGGCTGCAC |
| *Runx1* | F: CACCGTCATGGCAGGCAAC<br>R: GGTGATGGTCAGAGTGAAGC |
| *Actin, beta* | F: GACCTCTATGCCAACACAGT<br>R: AGTACTTGCGCTCAGGAGGA |
| *Cebpb* | F: GTTTCGGGACTTGATGCAAT<br>R: CCCCGCAGGAACATCTTTA |
| *Cebpe* | F: AGTACCAAGTGGCACACTGC<br>R: GAGAAGGGGACTGCAGGGA |
| *c-Fos* | F: CCTACTACCATTCCCCAGCC<br>R: CTGTCACCGTGGGGATAAAG |
| *c-Jun* | F: GAAAAGTAGCCCCCAACCTC<br>R: AATCAGACAGGGGACACAGC |
| *Ets1* | F: AGCCGACTCTCACCATCATC<br>R: CAAGGCTTGGGACATCATTT |
| *Gfi1* | F: TCCCTGTCAGTACTGTGGCA<br>R: TGGAGCTCTGACTGAAGGCT |
| *Gfi1b* | F: CTCATAACGTTGACCGAGCC<br>R: AAGGACCGTGGCATTTTTC |
| *JunB* | F: ATCCCTATCGGGGTCTCAAG<br>R: AGGCTAGCTTCAGAGATGCG |
| *Klf1* | F: CTTTGGCACCTAAGAGGCAG<br>R: CAGGAGCAGGCATAAGGC |
| *Klf4* | F: AAAAGAACAGCCACCCACAC<br>R: CGTCCCAGTCACAGTGGTAA |
| *Klf5* | F: GTAACCCGGATCTGGAGAAG<br>R: CAGGTGCACTTGTAGGGCTT |
| *Meis1* | F: TCCACTCGTTCAGGAGGAAC<br>R: CTGGGGGAAGCTACACTGTT |
| *Tal1/SCL* | F: AACAACAACCGGGTGAAGAG<br>R: CATTCACATTCTGCTGCCTC |
| *Cebpg* | F: GCGCAGAGAGCGGAACAA<br>R: GTATCTTGAGCTTTCTGCTTGCT |
| *CEBPA* | F: CGGTGGACAAGAACAGCAAC<br>R: CGGAATCTCCTAGTCCTGGC |
| *ACTB* | F: CATGTACGTTGCTATCCAGGC<br>R: CTCCTTAATGTCACGCACGAT |
