# Supplementary material for: Granulopoiesis Requires Increased C/EBPα Compared to Monopoiesis, Correlated with Elevated Cebpa in Immature G-CSF Receptor versus M-CSF Receptor Expressing Cells
Source: PLoS One. 2014 Apr 21;9(4):e95784. doi: 10.1371/journal.pone.0095784 (PMC3994156; doi:10.1371/journal.pone.0095784)
Supplement: Table S2 — Fold-change (FC) of indicated RNAs by Cebpa B9 shRNA vs vector in lineage-negative marrow cells. (PDF) [file pone.0095784.s006.pdf]

TABLE S2. Fold-change (FC) of indicated RNAs by *Cebpa* B9 shRNA vs vector in lineage-negative marrow cells										
**SYMBOL**	**FC Exp 1**	**FC Exp 2**		**SYMBOL**	**FC Exp 1**	**FC Exp 2**		**SYMBOL**	**FC Exp 1**	**FC Exp 2**
Aatk	-2.78606	-2.1959		Bcl11a	1.773192	1.668057		Ctsb	-1.610444	-1.624736
Abcb4	3.473837	2.83297		Blvrb	1.856152	1.892175		Ctsg	-1.942708	-1.698496
Abcd1	-1.58644	-1.78119		Bzrap1	-2.47846	-2.27463		Ctsh	-1.923585	-1.790923
Abcd2	-1.69447	-1.59032		C3	-3.36181	-3.57088		Cxcr4	-1.717749	-1.58807
Abhd5	-2.6176	-2.10194		Camp	-7.71809	-8.27151		Cyba	-1.450878	-1.463026
Abi3	2.313458	2.088759		Capn5	2.223086	2.36851		D3Ucla1	-1.526507	-1.430294
Acadsb	-1.67383	-1.60237		Car1	1.589696	1.593206		Dcakd	1.737637	1.569008
Acot1	-2.11445	-2.24388		Car12	1.848505	1.635572		Ddit4	1.902778	1.682345
Acpl2	-2.13806	-1.96787		Car13	1.419987	1.618723		Dgat2	-7.612098	-4.988847
Acpp	-1.87856	-1.46731		Car2	4.364771	4.190226		Dgkg	-1.956478	-1.969725
Acsl1	-1.74634	-1.73664		Casz1	-2.63495	-2.18761		Dhrs7	-1.61073	-1.578228
Acss1	2.444768	2.429952		Ccdc125	-1.48326	-1.89977		Dhrs7b	1.503774	1.465265
Actn1	-1.86674	-1.76072		Ccl6	-7.1412	-3.19703		Dmkn	-3.428072	-2.997608
Adfp	-1.49775	-1.84183		Ccnd1	4.985514	3.633958		Dnajc6	1.615083	1.443701
Adssl1	-2.00096	-1.88669		Ccno	-3.0429	-2.4109		Dnmt3b	2.467118	2.042879
Aff1	-1.51449	-1.4831		Cd177	-2.18462	-1.80093		Dnmt3l	-2.840231	-2.391078
Agpat2	-1.79333	-1.41865		Cd27	1.551881	1.446977		Dok2	1.54764	2.293956
Agps	-3.49333	-2.97619		Cd34	1.758961	1.670466		Dok3	-2.412002	-2.045307
Agtrap	-1.53495	-1.62834		Cd52	4.140935	2.67311		Dolpp1	-1.661132	-1.542562
Akr1c18	-6.83423	-2.86504		Cd59a	2.003008	2.842894		Dpp4	2.591039	2.437946
Alad	2.030576	2.069589		Cd72	1.661972	1.695804		Drctnnb1a	1.426041	1.59152
Alas1	-2.58051	-1.94711		Cd74	1.664893	2.076412		Dstn	-3.477062	-2.687704
Aldh3b1	-1.66829	-1.65585		Cd93	-2.01649	-2.20717		Dtx4	-1.954056	-1.591283
Aldh4a1	-1.88349	-1.83244		Cd96	1.45	1.412989		Dusp1	1.828865	1.549144
Als2	1.921425	2.194735		Cda	2.572719	2.108132		Dusp16	1.641319	1.456615
Angpt1	1.498685	1.862396		Cdkn1a	1.612732	1.635055		Dyrk3	1.488968	1.569273
Ank	1.560758	1.544044		Ceacam1	-1.81092	-1.41611		Ear2	-3.132222	-2.543087
Ankrd28	-1.74319	-1.59567		Ceacam2	-1.81679	-1.62636		Ear3	-2.228286	-1.784173
Anxa1	-4.28826	-2.41542		Cebpa	-1.87106	-1.74783		Ear4	-3.929922	-2.696113
Anxa2	-1.52583	-1.77534		Cebpe	-4.40164	-3.46027		Ear6	-7.791869	-3.110962
Anxa3	-4.521	-3.04478		Celsr3	-1.92403	-1.54056		Egfl7	1.612459	1.997446
App	-2.17113	-1.91233		Chchd10	-2.0676	-1.78575		Ela1	1.408222	1.509103
Aqp1	4.358209	3.558584		Chd7	-1.78746	-2.05863		Ela2	-9.972096	-5.61423
Aqp9	1.461252	1.975941		Chi3l1	-2.38816	-1.49906		Epb4.1l4b	1.729266	1.655452
Arhgef6	-1.57862	-1.53535		Chi3l3	-8.47139	-12.439		Epdr1	1.984454	2.230618
Arid3a	-2.44681	-1.91178		Chi3l4	-8.18977	-9.78813		Epx	-3.153058	-2.484468
Arl11	-3.33969	-2.42249		Chordc1	-2.76831	-2.10173		Eraf	3.707257	2.654888
Arsb	-2.72417	-2.21665		Chpt1	-2.11615	-1.91684		Ethe1	-1.952841	-1.55654
Art4	1.607731	1.630191		Chst12	-1.95853	-1.74717		Ets1	-2.952715	-1.800166
Atf5	-1.778	-1.99815		Chst13	-3.16766	-2.12042		F10	-3.340401	-2.62533
Atg12	-2.64751	-2.02606		Cldn15	-3.28275	-3.07503		F13a1	-2.877161	-4.742674
Atp13a2	1.516652	1.612843		Clec5a	-1.56804	-1.97578		F2r	1.791904	3.119444
Atp1b2	1.539749	1.429817		Cnih4	-1.51656	-1.56156		F2rl3	1.48705	1.533169
Atp6ap2	-1.72285	-1.59333		Cnn2	-1.73542	-1.4594		Fam102a	2.100278	2.353105
Atp6v0b	-1.62729	-1.45691		Col18a1	1.955182	2.210647		Fam129b	2.652116	1.892715
Atp8b4	-1.55338	-1.75678		Creld2	-2.53173	-1.74642		Fam160a2	-2.883949	-2.184552
B3gnt8	-1.8534	-1.94335		Cryl1	1.658901	1.503988		Fbxo21	1.40877	1.409892
B4galt6	-4.71611	-3.23629		Csf2ra	-1.45455	-1.6888		Fcgr2b	-1.98491	-1.625026
Bace1	-1.49544	-1.57676		Csf3r	-1.5004	-1.58565		Fcgr3	-3.208237	-2.242771
Bak1	-1.62706	-1.63885		Csnk1e	1.760719	1.772779		Fcgr4	-1.596765	-1.460989
Bambi-ps1	-2.59901	-1.75621		Cstb	-2.12218	-1.73885		Fcnb	-5.426349	-4.052279
Bat5	1.463544	1.406789		Ctdspl	-2.00622	-1.66687		Fgd4	-1.626046	-1.453322
Bcas1	-1.61913	-1.45384		Ctla2b	1.631841	1.94024		Fkbp11	-2.540422	-1.748762

SYMBOL	FC Exp 1	FC Exp 2		SYMBOL	FC Exp 1	FC Exp 2		SYMBOL	FC Exp 1	FC Exp 2
Flot2	-2.77876	-2.44469		Ier3	2.036012	1.755508		Mogat2	-2.190726	-1.82568
Fmnl2	-1.50309	-1.45437		Ifi47	1.403711	1.940877		Mpo	-2.23701	-1.818724
Fndc3b	-1.67803	-1.7823		Ifitm3	1.739295	1.473669		Mpp1	-2.394564	-1.613908
Frat2	-1.59222	-1.68036		Ifngr1	-1.82287	-1.92529		Mreg	-3.204831	-2.520615
Fut4	-1.48269	-1.50428		Ikbke	-1.74547	-1.46804		Mrps26	-1.759823	-1.574981
G0s2	-2.3362	-2.17605		Il1b	1.411448	1.906918		Ms4a3	-2.984152	-2.199459
G6pdx	-1.43378	-1.48318		Imap38	1.501224	1.664406		Mta3	1.946294	2.054077
Gadd45a	-1.90758	-1.84293		Ipo4	1.61443	1.546884		Mtap	-2.57909	-1.986525
Gapt	-3.7969	-2.88921		Irak3	-1.89398	-1.51178		Mtf1	-1.567849	-1.497649
Gca	-2.72821	-2.38961		Irf2	1.511095	1.458961		Mtus1	-1.811791	-1.964861
Gcnt2	1.435876	1.487617		Irf9	1.595868	1.628871		Muc13	1.730139	1.420766
Gda	-1.47577	-1.4087		Itgb2l	-1.57054	-1.56654		Myl4	1.673523	1.866586
Gfi1	-4.95707	-3.65471		Itpripl2	-1.41541	-1.50632		Mylc2pl	-2.814303	-2.470598
Gfi1b	3.697187	3.558674		Kcnab2	-1.49196	-1.47809		Myo1f	-1.681348	-1.759078
Gga2	-2.42195	-1.76988		Kcnn4	1.868408	1.745331		Naip2	-1.430607	-1.678168
Gimap1	2.608771	3.499111		Klf1	2.618367	2.342917		Nalp6	3.311192	3.411313
Gimap6	1.453445	1.773497		Lat2	1.40766	2.020919		Narf	1.518424	1.428734
Gimap9	1.575174	1.483636		Lbp	-2.62437	-2.91596		Necap2	1.546315	1.445479
Gjb3	-1.78133	-1.7933		Lcn2	-4.12649	-5.99512		Nedd9	-1.753119	-1.685875
Glrx	-1.66848	-1.6454		Lgals3	-5.27795	-3.93985		Neu1	-1.71897	-1.790033
Glrx1	-1.55063	-1.64828		Lgals3bp	2.537506	2.323946		Nfe2l2	-1.549458	-1.412781
Gmpr	2.019369	2.246991		Lgals9	1.61514	1.874464		Nfkbia	1.427779	1.505694
Gna14	1.5199	1.527914		Lincr	1.4858	1.593681		Ng23	2.54024	2.516209
Golm1	2.343428	2.088784		Lmf1	-1.4381	-1.4424		Ngp	-15.67212	-22.59197
Gpc1	-2.13735	-2.38823		Lmna	2.678271	2.762077		Nin	-2.035007	-1.86016
Gpi1	-1.46067	-1.42143		Lpl	1.436709	-1.43671		Nipa1	1.531404	1.543454
Gpr128	1.662515	1.502521		Lpxn	2.146779	1.978042		Nkg7	-1.796487	-1.897756
Gpr160	-2.90839	-2.14402		Lrg1	-2.93872	-2.61374		Nod1	-1.669549	-1.886353
Gpr177	-2.04607	-1.57851		Lrp12	1.796386	1.627343		Nras	-2.32673	-1.81336
Gpr84	-1.6081	-1.67436		Lrrc8d	-1.4768	-1.44875		Nrp1	-2.388553	-1.501567
Gpx3	-1.77071	-1.9202		Ltb	1.772461	1.782787		Nt5dc2	-1.500683	-1.408352
Grcc10	1.704474	1.640522		Ltb4r1	-4.19765	-3.2416		Nuak2	-1.690133	-1.479658
Gria3	-1.50316	-1.60159		Ltc4s	1.577017	1.454623		Nudt7	1.427564	1.800152
Gstm1	-5.12172	-3.8106		Ltf	-3.12841	-1.96164		Olfml2b	-2.251902	-1.908305
Gyg	-1.65856	-1.58297		Ly6a	4.467999	4.043531		Ormdl3	1.672383	2.02604
Gypa	1.765562	1.676686		Ly6c1	-7.65394	-6.29582		Osbpl3	-1.573529	-1.486243
H2-Q8	1.593446	2.179857		Ly6e	1.560928	1.536854		Ostm1	-1.575304	-1.409141
H47	-1.5102	-1.41387		Lyrm5	-1.67927	-1.51314		P2ry1	-1.569405	1.531944
Hba-a1	21.8606	15.99266		Lyz	-1.46101	-1.958		P4hb	-1.498463	-1.413801
Hbb-b1	5.250435	5.371228		M6prbp1	-2.1034	-1.83471		Pabpc4	-1.566113	-1.515253
Hdc	-3.48577	-1.71637		Map3k11	-1.8685	-1.73819		Pacs1	1.409157	1.583395
Hdlbp	-1.75798	-1.57863		Map3k3	-1.84457	-1.60401		Pacs2	1.750809	1.433943
Heatr5a	1.509398	1.538639		Map3k6	1.683646	1.587019		Palm	-1.659871	-1.568807
Hebp1	1.779929	1.574527		Mapkapk3	-1.44782	-1.44857		Papss2	-2.349016	-1.681653
Hectd1	1.49349	1.572807		Mcee	-1.9867	-1.80684		Pcyt1b	1.623964	2.043536
Hemgn	2.671083	2.343039		Mcfd2	-1.85879	-1.57051		Pdia4	-1.837615	-1.524305
Hgf	-2.48642	-1.55697		Mcpt8	-4.5473	-2.37907		Pdlim1	-2.201746	-1.73681
Hgfac	2.275188	2.052899		Med21	-2.14657	-1.60888		Pfkfb4	-2.551391	-2.079286
Hmgn3	-6.5172	-2.0527		Meis1	1.687968	1.74102		Pgd	-1.700537	-1.638297
Hp	-2.90315	-4.08923		Mettl9	-1.96191	-1.93159		Pglyrp1	-4.21329	-4.168944
Hs6st1	1.491117	1.478051		Mfge8	2.052709	2.0615		Phc1	1.510388	1.520448
Hsd11b1	-4.81155	-4.68056		Mgat4b	1.45433	1.418801		Phlda1	1.470159	1.708334
Hsd17b1	-2.53137	-2.28518		Mgst1	-3.73642	-3.25582		Phlda2	3.838898	3.049587
Hvcn1	-3.24043	-3.10477		Mgst2	-3.00975	-3.02405		Pik3ap1	-1.499485	-2.00227
Idh1	-1.70979	-1.78512		Mist1	-1.53219	-1.50599		Pip4k2a	1.90561	1.598422

SYMBOL	FC Exp 1	FC Exp 2		SYMBOL	FC Exp 1	FC Exp 2		SYMBOL	FC Exp 1	FC Exp 2
Pld1	-1.89286	-1.55805		Sepx1	-2.75381	-2.26428		Thbs1	-2.891735	-1.98036
Plek	2.045833	2.177696		Serpina3f	2.389708	2.865986		Tlr4	-1.474755	-1.561682
Plod3	-2.03322	-1.77076		Serpina3g	1.40255	2.204494		Tm6sf1	-1.562121	-1.636923
Plscr1	2.70499	2.93983		Serpinb1a	-2.04432	-1.49638		Tmc7	-1.557761	-1.442654
Plxnd1	-2.03559	-1.85137		Serpine2	-1.83545	1.779317		Tmed3	-2.787634	-1.85337
Podxl	1.463403	2.180858		Serpinf1	2.024174	1.441073		Tmem109	2.296614	1.498087
Ppapdc1	-2.21944	-1.89435		Sesn1	1.485896	1.692223		Tmem176b	2.320219	2.295245
Ppapdc1b	-2.74967	-1.89118		Sgk1	-2.42316	-1.62613		Tmem180	-1.471889	-1.431241
Ppic	-2.62513	-1.82232		Sgms2	-2.05104	-1.82392		Tmem23	1.477374	1.722192
Ppif	1.616629	1.674081		Sgpp1	-2.01643	-1.89601		Tmem38b	-2.155247	-2.101419
Ppm1m	-1.58393	-1.55871		Sh3kbp1	-1.61469	-1.47057		Tmem39a	-1.684802	-1.596977
Ppp1r13b	1.487214	1.462436		Sh3tc1	-1.61727	-1.50402		Tmem50b	-2.343654	-2.163105
Pqlc3	-1.64098	-1.46565		Sipa1l1	1.467854	1.510809		Tmem53	-1.495552	-1.537587
Prdx4	3.367898	2.476497		Sirpa	-1.79838	-1.98618		Tnf	1.58887	1.596023
Prdx5	-1.92734	-1.54469		Sla2	3.148479	2.665422		Tnfrsf21	-1.72088	-1.407482
Prg2	-4.29357	-3.62427		Slc14a1	1.617921	3.202525		Tnfrsf26	-1.73743	-1.567689
Prg3	-3.25	-2.10024		Slc16a6	-1.44561	-1.60121		Tnfsf12-tnfsf13	-2.3433	-2.553554
Prkcb	-1.90047	-1.64971		Slc22a4	-1.68644	-1.52681		Tnik	1.435897	1.586572
Prnp	1.78447	2.439533		Slc24a3	1.551007	2.176899		Tpcn1	-1.529356	-1.518984
Prom	-1.51957	-1.40177		Slc24a6	-3.22413	-2.58346		Tpst1	1.581051	1.400079
Prss34	-47.6318	-19.3186		Slc28a2	-1.56539	-1.46948		Trem3	-3.812979	-3.41592
Prssl1	-2.23434	-1.90099		Slc2a6	1.857682	1.530962		Trim11	-1.401282	-1.427149
Prtn3	-2.69641	-2.2273		Slc38a5	3.479519	2.321033		Trim45	-1.450249	-1.443764
Ptp4a3	1.548473	1.651938		Slc39a4	1.613323	1.703302		Trip6	1.404133	1.555556
Pygl	-2.26853	-1.92489		Slc40a1	-3.5145	-3.39848		Trove2	1.417271	1.568256
Rab27a	-2.62647	-2.09448		Slc43a1	1.560904	1.532378		Tsc22d1	1.562874	1.517373
Rab31	-1.54251	-1.44144		Slc45a3	1.804612	2.268173		Tspan33	1.797449	1.982536
Rab32	-1.8322	-1.97057		Slpi	-5.9626	-3.09279		Tubb6	-1.414219	-1.699348
Rab3d	-2.20818	-1.95564		Smap2	2.205949	1.977287		Txndc13	-6.476911	-4.086424
Ralb	-1.90165	-1.64858		Smpdl3a	-1.59453	-1.70257		Uap1	-1.559767	-1.492497
Rasa2	-1.50894	-1.49711		Snai3	-1.99206	-1.80389		Ube1l	1.465237	2.28104
Rasgrp2	-1.80122	-1.51147		Snap23	1.606787	1.627971		Unc84b	-2.751178	-2.092582
Rbms1	-1.60323	-1.60041		Snx18	-1.5043	-1.66577		Usp18	2.611264	3.234808
Rbpms	1.412564	1.529259		Sorl1	-2.82485	-3.33019		Vamp5	1.891608	2.178572
Rfesd	1.428335	1.480512		Sort1	-3.32373	-2.41021		Vamp8	1.403484	1.527022
Rftn1	1.428962	1.506347		Sox4	1.662055	1.455767		Vat1	-4.256589	-2.361801
Rgs1	1.793987	1.950537		Spp1	-1.77812	-2.50861		Vcam1	-1.494981	-1.511753
Rhob	-1.53661	-1.64231		Spred1	2.03734	1.511286		Vezf1	1.473708	1.651634
Rhou	-2.18696	-1.85838		Srgn	-1.95555	-1.54861		Vim	-1.461063	-1.406327
Rnase12	-5.29757	-2.5519		Ssbp3	1.530606	1.472962		Xbp1	-2.186884	-1.815985
Rnd3	-1.52403	-1.61862		St3gal5	1.90581	1.907586		Xdh	-1.513426	-1.564627
Rsad1	1.464359	1.538267		St3gal6	1.923907	2.244531		Xlr4a	1.669714	1.47084
S100a11	-2.19039	-2.21917		Stab1	-1.61121	-1.43001		Xpc	-1.659959	-1.452587
S100a8	-2.81365	-11.1406		Stfa1	-2.21681	-15.316		Ybx3	1.482511	1.483955
S100a9	-6.5495	-33.0747		Susd3	1.605244	1.754553		Ypel3	1.646499	1.553087
Samd14	2.158168	2.698607		Syngr2	-1.50185	-1.45906		Ypel5	-2.227888	-1.781476
Scamp4	-2.12782	-1.90224		Tal1	1.810074	2.205297		Zfp36l1	1.791717	1.612261
Sccpdh	-1.5725	-1.62579		Tank	-1.44849	-1.68964				
Scin	2.181513	3.351993		Tbc1d2	-1.67622	-1.41027				
Sdcbp	-1.76034	-1.59498		Tceb3	-1.66339	-1.48827				
Sdf2l1	-1.81854	-1.47414		Tcirg1	-1.44755	-1.48945				
Sdsl	1.772752	2.133542		Tcn2	-2.29463	-2.16339				
Selplg	-1.51957	-1.56288		Tex2	-2.40975	-1.97583				
Sema4a	-3.33802	-3.02891		Tex9	1.677935	1.68186				
Sepn1	-1.6219	-1.59078		Tgm2	-1.59541	-1.88078				
